# Time-ordered co-expression network analysis reveals *b*-locus-mediated transcriptional cascades underlying sugarcane resistance to smut fungus (*Sporisorium scitamineum*)

**DOI:** 10.3389/fpls.2026.1929311

**Published:** 2026-10-16

**Authors:** Xiu-Peng Song, Xiao-Qiu Zhang, Bing-Qiang Lu, Dong-Mei Huang, Yong-Jian Liang, Bao-Qing Zhang, Yu-Chi Deng, Dong-Mei Li, Yang-Rui Li, Qiang Liang, Krishan K. Verma, Mei-Xin Yan

**Affiliations:** 1 Sugarcane Research Institute, Guangxi Academy of Agricultural Sciences/Key Laboratory of Sugarcane Biotechnology and Genetic Improvement (Guangxi), Ministry of Agriculture and Rural Affairs/Key Laboratory of Sugarcane Genetic Improvement (Guangxi), Nanning, Guangxi, China; 2 Guangxi South Subtropical Agricultural Science Research Institute, Chongzuo, Guangxi, China

**Keywords:** b-locus, hormone hijacking, JA/ET signaling pathway, *Sporisorium scitamineum*, sugarcane smut, TO-GCN

## Abstract

Sugarcane smut, caused by the biotrophic fungus *Sporisorium scitamineum*, causes serious yield and sugar losses. Growing resistant varieties is the main control method. The fungal b mating-type locus (*b*-locus) is required for filamentous growth and virulence, but how it shapes the host response is not well understood. Here, we compared a resistant cultivar (GT29) and a susceptible cultivar (GT11-80) after inoculation with a wild-type (WT) strain or a *b*-locus deletion mutant, with sampling from 0 to 7 days postinoculation (dpi). We measured bE gene copy number by quantitative PCR (qPCR), assayed defense enzymes and phytohormones, and performed RNA sequencing followed by time-ordered gene co-expression network (TO-GCN) analysis. The WT strain proliferated rapidly in GT11-80 but was strongly restricted in GT29, and the *b*-locus mutant reached much lower levels than the WT strain in GT11-80 by 7 dpi. GT29 showed a stronger and more sustained oxidative burst (O_2_^−^ and H_2_O_2_) than GT11-80 and maintained higher antioxidant enzyme activities. For hormones, WT infection induced more jasmonic acid (JA) and 1-aminocyclopropane-1-carboxylic acid (ACC; the ethylene precursor) than the mutant, with the highest levels in GT29 at 5 dpi, whereas salicylic acid (SA) accumulated late (7 dpi) in GT11-80; abscisic acid (ABA) stayed high under WT infection but dropped under the mutant. TO-GCN analysis identified a WT-specific, time-ordered transcription factor (TF) cascade in GT11-80 that was lost under mutant infection, whereas the GT29 network kept its temporal order under both conditions. In the jasmonic acid/ethylene (JA/ET) subnetwork, *ERF1* was a central hub in GT11-80, while in GT29 *ERF1* was much less connected and *EIN3* was the main hub. Overall, the *b*-locus is required for the time-ordered transcriptional response during infection, and the resistant and susceptible cultivars differ in their hormone dynamics and JA/ET network structure. These findings provide candidate genes and network features for breeding smut-resistant sugarcane.

## 1 Introduction

Sugarcane smut is caused by *Sporisorium scitamineum* and is one of the most destructive sugarcane diseases worldwide. This biotrophic fungus lowers cane yield and reduces sucrose quality and is a serious threat to sugarcane production (Rajput et al., 2021, 2022). The pathogen spreads through airborne teliospores and infected seed cane. The infection becomes systemic and produces the typical “whip” symptom. Fungicidal treatment of seed cane reduces smut development but gives only partial protection (Rajput et al., 2019), so the main control method is to breed and plant resistant cultivars, which is the most economical and sustainable strategy (Su et al., 2016). Therefore, understanding how *S. scitamineum* causes disease and how the host resists it is important for protecting sugarcane yield and quality.

An important virulence factor in smut fungi is the b mating-type locus, which controls mating and the change in cell shape needed for infection. In the corn smut model *Ustilago maydis*, the *b*-locus encodes two homeodomain proteins (bE and bW). When two compatible haplotypes meet, these proteins form a heterodimer that turns on a transcriptional program for filamentous growth and pathogenic development (Wallen et al., 2021). This *b*-factor is required to build the dikaryotic infection structure and to let the fungus grow inside the host. In *S. scitamineum*, the *b* locus is also needed for sexual mating, filamentous colony growth, and virulence (Yan et al., 2016). If either part of the b heterodimer is disrupted, the fungus cannot form whips and disease is strongly reduced. These results indicate that the *b*-locus controls pathogenic development, so it is a good target for studying the molecular basis of virulence.

Using mutant strains to study virulence gene function is a common approach in plant pathogen research. For example, a pep1 mutant of *U. maydis* caused a strong early defense reaction in maize, including an oxidative burst with high H_2_O_2_ and strong induction of a maize peroxidase gene (POX12); these responses were mostly absent during wild-type infection (Hemetsberger et al., 2012). These results showed that Pep1 suppresses host defenses, and other work showed that Pep1 directly inhibits host peroxidase activity to block ROS-based immunity. A similar case was reported for Xanthomonas. An avrb6-deletion strain did not induce GhSWEET10 and caused much milder symptoms. Wild-type bacteria carrying avrB6 strongly induced GhSWEET10, which showed that Avrb6-driven transcriptional activation is a key virulence mechanism (Cox et al., 2017). Such comparisons show how single pathogen genes change host immune responses and help define their role in host defense.

The host response is another important factor, and it varies a lot among sugarcane varieties. Cultivars differ in their basic resistance, which often shows up as differences in how fast the pathogen spreads and how strongly defenses are activated (Chen et al., 2023). Large-scale germplasm screening has shown that smut incidence ranges from immunity to more than 50% among cultivars and that infection of susceptible cultivars reduces sett germination and increases tillering (Rajput et al., 2024). For example, GT29 is highly resistant and shows strong defensive protein and enzyme responses. Susceptible cultivars such as Yacheng71-374 and ROC22 allow heavy fungal colonization (Chen et al., 2023; Singh et al., 2021). Earlier studies report that resistant varieties build up more phenolic compounds and pathogenesis-related proteins to limit fungal growth (Chen et al., 2023). Susceptible hosts allow faster pathogen growth and suffer more tissue damage (Su et al., 2016). By comparing a resistant genotype (GT29) with a susceptible one (GT11-80), we tried to capture these differences and to test how the *b*-locus affects the course of defense. With this design, using a virulent and an attenuated pathogen on resistant and susceptible hosts, we could compare host responses associated with WT and b-locus-deficient strains in resistant and susceptible genetic backgrounds.

In this study, we followed the infection process of *S. scitamineum* with and without a functional *b*-locus in two sugarcane cultivars and looked at how pathogen and host factors work together. This let us see how the *b*-locus controls the fungal infection program and how the host defense network responds in the resistant and the susceptible background. The results help explain the mechanism of sugarcane smut and provide information for breeding durable resistance.

## 2 Materials and methods

### 2.1 Plant materials and infection methods

Single-bud segments of the resistant sugarcane cultivar GT29 (Singh et al., 2021) and susceptible cultivar GT11-80 (Duan et al., 2023) were sourced from the Sugarcane Research Institute of the Guangxi Academy of Agricultural Sciences (China). All materials were verified to be free of *Sporisorium scitamineum* prior to challenge. Bud segments were excised from uniform, healthy stalks, trimmed to one-bud units, and pretreated in a 52°C water bath for 30 min to promote germination. After cooling, segments were surface-sterilized in 0.01% sodium hypochlorite for 10 min, rinsed three times in sterile distilled water, and placed on trays layered with sterilized vermiculite in a growth chamber (32°C, 12 h light/12 h dark) until the emerging shoot reached ~2 cm. Two fungal inocula were prepared: (1) a highly virulent wild-type mixture (MAT-1 + MAT-2) and (2) a laboratory-constructed, *b*-locus deletion mutant (SsΔMAT-1b + MAT-2), which is defective in whip formation and exhibits dramatically reduced pathogenicity (Yan et al., 2016). Mixed suspensions were incubated at room temperature for 30 min to allow mating and then adjusted to 5 × 10^6^ spores mL^−1^. For inoculation, each bud segment was injected at the basal meristem with 100 µL of the appropriate mating suspension until a small droplet emerged at the shoot tip; mock controls received 100 µL sterile water as a procedural background. All treatments, including the mock treatment, were injected with the same volume through the same procedure to ensure that the mechanical wounding caused by injection was identical in every treatment. All the physiological, biochemical, and transcriptome data reported in this study were therefore obtained only from plants inoculated with the WT strain or with the b-locus mutant strain, and all comparisons were made between these two strains within each cultivar. Inoculated segments were returned to vermiculite trays under the same growth conditions.

Samples were collected at 0, 1, 3, 5, and 7 days postinoculation (dpi). At each time point, five bud segments per treatment were pooled to form one biological replicate, with three replicates per treatment. The 0-dpi samples were collected within 30 min after inoculation. All plants were inoculated in a single batch under identical conditions, and at each time point, the inoculated plants of a given treatment were divided into three groups of five bud segments; the segments within each group were pooled to give one replicate, yielding three replicates per treatment and time point. Harvested tissues were briefly blotted to remove surface liquid, flash-frozen in liquid nitrogen, and stored at –80°C until extraction. The tissue harvested at each time point was the basal meristem region of the bud segment, which is the tissue surrounding the inoculation site. The same tissue was used for all analyses reported in this study.

### 2.2 Quantification of *S. scitamineum*

Real-time quantitative PCR (qPCR) was performed to quantify *S. scitamineum* infection levels. Genomic DNA was extracted from 0.1 g of sugarcane basal meristem tissue using the BioFastSpin Plant genome extraction kit (Bioer, China) following the manufacturer’s instructions. The qPCR targeted the bE gene of *S. scitamineum* using primers bEQ-F and bEQ-R designed from GenBank accession U61290.1 (Su et al., 2013). The b-locus deletion mutant (SsΔMAT-1b) was constructed by deleting the entire b mating-type locus (including both bE and bW) in the MAT-1 haploid strain (Yan et al., 2016). The mutant dikaryon was formed by crossing this MAT-1 Δb-locus haploid with a compatible MAT-2 haploid, which retains an intact bE gene. The qPCR target sequence is present in both WT and mutant dikaryons. Because the primer binding sites are identical in the two strains, the amplification efficiency is the same; however, the wild-type dikaryon carries two bE alleles per cell (MAT-1 and MAT-2) whereas the mutant dikaryon carries only one (MAT-2). The values are therefore reported as bE gene copy number per gram of tissue and should not be interpreted as strictly equivalent fungal biomass between the two strains. Because the mutant carries at most half as many bE copies per fungal genome as the wild type, differences of less than about twofold between the two strains cannot be interpreted as differences in colonization; comparisons over time within a strain are not affected by this offset. Each 12.5-μL reaction contained 1.0 μL of template DNA, 1.0 μL each of 10-μM primers (bEQ-F and bEQ-R), and 1× ChamQ Universal SYBR qPCR Master Mix (Vazyme, China). The PCR program was: 95°C for 3 min, followed by 40 cycles of 95°C for 30 s, 58°C for 30 s, and 72°C for 20 s, followed by melting-curve analysis from 60°C to 95°C. A single peak in the melting curve confirmed the specificity of the amplification, and the identity of the amplicon was further verified by cloning and Sanger sequencing. Reactions were run in technical duplicates using a Roche LC480 Real-Time PCR System (Roche, Switzerland). bE gene copy number was determined by absolute quantification based on a standard curve generated from 10-fold serial dilutions of a plasmid containing the *bE* gene fragment (Su et al., 2013). Statistical comparisons were performed using a t-test (p < 0.05). The full sequences of primers (bEQ-F: 5′-TGAAAGTTCTCATGCAAGCC-3′; bEQ-R: 5′-TGAGAGGTCGATTGAGGTTG-3′) are listed in **Supplementary Table 7**. The standard curve was linear over six 10-fold dilutions (2.93 × 10³ to 2.93 × 10^8^ copies µL^−1^), with a slope of −3.5114, a correlation coefficient (R²) of 0.9982, and an amplification efficiency of 92.7%.

### 2.3 Quantification of reactive oxygen species

The contents of superoxide anion (O_2_^−^) and hydrogen peroxide (H_2_O_2_) were determined from 0.1 g of frozen basal meristem tissue at each time point using commercial assay kits (Solarbio, Beijing, China). Superoxide anion content was measured using a superoxide anion assay kit (BC1290), and hydrogen peroxide content was measured using a hydrogen peroxide assay kit (BC3590). Tissue was ground in liquid nitrogen, homogenized in the ice-cold extraction solution provided with each kit, and processed according to the manufacturer’s instructions. O_2_^−^ was measured from the absorbance at 530 nm and H_2_O_2_ from the absorbance at 415 nm, using a microplate reader Epoch (Biotek, USA). Three biological replicates were analyzed per treatment and time point.

### 2.4 Determination of defense-related enzyme activities

Enzyme activities were assayed using crude extracts prepared from 0.5 g of frozen basal meristem tissue, ground in liquid nitrogen, and homogenized in 5 mL of ice-cold extraction buffer (50 mM phosphate buffer, pH 7.0, containing 1 mM EDTA, 1% polyvinylpyrrolidone, and 0.1% Triton X-100). Homogenates were centrifuged at 12,000 × g for 15 min at 4°C, and the supernatant was used for all assays. The activities of β-1,3-glucanase (BC0360), phenylalanine ammonia-lyase (PAL, BC0210), superoxide dismutase (SOD, BC0170), catalase (CAT, BC0200), peroxidase (POD, BC0090), ascorbate peroxidase (APX, BC0220), and chitinase (BC0820) were measured with commercial assay kits (Solarbio, Beijing, China) according to the manufacturer’s instructions, using a microplate reader Epoch (Biotek, USA). Three biological replicates were analyzed per treatment and time point. All activities were calculated on a tissue fresh-weight basis and are reported as units per gram of tissue (U g^−1^). The reaction principle, the detection wavelength, and the definition of one unit of activity for each kit are given in **Supplementary Table 10**.

### 2.5 Quantification of phytohormones

The contents of gibberellic acid (GA_3_), abscisic acid (ABA), jasmonic acid (JA), indole-3-acetic acid (IAA), salicylic acid (SA), and 1-aminocyclopropane-1-carboxylic acid (ACC) were measured at each time point. About 100 mg of frozen basal meristem tissue was ground to a fine powder in liquid nitrogen and extracted with 1 mL of cold methanol containing 1 mM butylated hydroxytoluene at 4°C for 16 h, following a previously described method (Luo et al., 2023). The extracts were analyzed on an ultra-high-performance liquid chromatography (UHPLC)-triple quadrupole mass spectrometry system consisting of an Agilent 1290 Infinity UHPLC and an Agilent 6460 triple quadrupole tandem mass spectrometer (Agilent, California, USA), with instrument parameters set as described previously. Hormones were detected in multiple-reaction-monitoring (MRM) mode using the quantifier transitions (Q1/Q3, m/z) 345/143.1 (GA_3_), 263.1/153.1 (ABA), 176.1/129 (IAA), 209.2/58.8 (JA), 137.0/93.0 (SA), and 102.15/56.05 (ACC). Quantification was based on external standard curves prepared from standard solutions of each analyte, covering 1–500 μg/L for GA_3_, 1–1,000 μg/L for ABA, 0.1–10 μg/L for IAA, 1–100 μg/L for JA, 1–100 μg/L for SA, and 1–200 μg/L for ACC. Standard curves were generated with MassHunter software (Agilent, California, USA). Three biological replicates were analyzed per treatment and time point. The limits of detection and quantification were determined at signal-to-noise ratios of 3 and 10, respectively, and the recoveries of the six analytes ranged from 65.9% to 109.5%. All hormone concentrations reported in this study were above the corresponding limit of quantification, the lowest group mean being at least 3.0 times the limit of quantification for every analyte. The quantification, the recoveries, and the corresponding measured ranges are summarized in **Supplementary Table 9**.

### 2.6 RNA extraction, library construction, and Illumina sequencing

For RNA sequencing, basal meristem samples from both cultivars (GT29 and GT11-80) inoculated with either the wild-type or the *b*-locus mutant strain were collected at 0, 1, 3, 5, and 7 dpi, with three biological replicates per treatment and time point, yielding 60 libraries in total.

Total RNA was extracted using TRIzol reagent (Invitrogen), and RNA quality was assessed using an Agilent 2100 Bioanalyzer (RNA Integrity Number > 7). Libraries were constructed using the NEBNext Ultra RNA Library Prep Kit, including mRNA purification, fragmentation, cDNA synthesis, adapter ligation, and PCR enrichment. All 60 libraries were prepared and sequenced in a single batch, so that no batch factor was introduced into the design. Sequencing was performed on an Illumina NovaSeq 6000 platform (150-bp paired-end reads). Raw reads were quality-filtered with fastp v0.24.0 (-q 20 -u 30 -n 10 -l 15), which retained on average 97.9% of the reads and yielded a mean of 43.4 million clean reads (6.45 Gb) per library, with mean Q20 and Q30 values of 97.9% and 93.8% and a mean GC content of 54.5%. Clean reads were aligned to the sugarcane reference genome (Saccharum spp. hybrid R570) with HISAT2, giving a mean mapping rate of 89.6%, of which 78.9% of the mapped reads were located in exonic regions, and gene expression was quantified with StringTie. Pearson correlation coefficients between biological replicates were high (mean r = 0.948, range 0.904-0.960) and clearly higher than the correlation between different treatment groups (mean r = 0.799), and principal component analysis separated the libraries primarily by cultivar (PC1 = 23.3%; PC2 = 7.1%). The quality-control and mapping statistics of each library are listed in **Supplementary Table 8**, and the correlation heat map, the principal component analysis, and the distribution of replicate correlations are shown in **Supplementary Figure 3**. Differential expression and time-ordered gene co-expression network (TO-GCN) construction followed Chang et al. (2019), using the Pearson correlation for co-expression pairs.

### 2.7 Construction of the time-ordered gene co-expression network

Gene expression was quantified as fragments per kilobase of transcript per million mapped reads (FPKM), and transcription factors (TFs) were identified by annotation against PlantTFDB. The TO-GCN was constructed as described by Chang et al. (2019). For each cultivar, Pearson correlation coefficients were calculated for all TF–TF pairs separately under wild-type (W) and mutant (M) infection. For each condition, the correlation was computed across all 15 RNA-seq libraries of that condition, corresponding to five time points (0, 1, 3, 5, and 7 dpi) with three biological replicates per time point, so that the replicate libraries rather than time-point means were used as the input observations. A pair was defined as positively co-expressed under wild-type infection (W+) when its Pearson correlation coefficient (PCC) exceeded a cultivar-specific cutoff of 0.93 in GT11-80 and 0.90 in GT29, and as uncorrelated under mutant infection (M0) when −0.5 ≤ PCC < 0.5. The resulting *b*-locus-dependent (W+/M0) pairs were used to build the network. The PCC cutoffs were not chosen arbitrarily but were determined for each cultivar with the Cutoff module of the TO-GCN package, which derives them from the distribution of all pairwise correlation coefficients in the corresponding data set (Chang et al., 2019). Each cutoff was taken as the correlation value beyond which the probability of exceeding it by chance fell below 0.05, so that only 4.07% of all TF–TF pairs in GT11-80 and 4.30% in GT29 were retained as positively co-expressed. The corresponding correlation distributions and the derivation of the cutoffs are shown in **Supplementary Figure 4**. The stability of the network to the choice of cutoff was assessed by rebuilding it at progressively more stringent cutoffs and comparing the resulting level assignments with the original ones (**Supplementary Figure 5**). The level assignment was also checked against the measured expression data: in both cultivars, the level of a transcription factor was correlated with the time at which its expression peaked under wild-type infection (**Supplementary Figure 2**). An ethylene-responsive factor (EREBP, SoffiXsponR570.02Dg300000), which showed the highest expression at the earliest sampling time point (0 dpi), was selected as the seed node. The remaining TFs were assigned to sequential levels by breadth-first search (BFS). Level-specific expression was displayed as z-score-normalized FPKM heatmaps. Network construction was performed with the TO-GCN scripts of Chang et al. (2019). The co-expression subnetworks were visualized with Cytoscape. To evaluate the sensitivity of the network hierarchy to seed node choice, alternative early-expressed TFs from network levels 2 and 3 were tested as alternative seed nodes. The level assignments were compared using Spearman’s rank correlation.

### 2.8 Functional enrichment and subnetwork analysis

For each network level, genes co-expressed with that level’s TFs were tested for Gene Ontology (GO) and Kyoto Encyclopedia of Genes and Genomes (KEGG) pathway enrichment using clusterProfiler, with all expressed genes as the background. Functional annotations were obtained from the R570 genome annotation. Terms with an adjusted p-value <0.05 were considered significantly enriched.

### 2.9 Statistical analysis

Data are presented as mean ± SD of three biological replicates. For pathogen quantification, differences between the wild-type and mutant strains at each time point were assessed by Student’s *t*-test (p < 0.05). The reactive oxygen species contents, enzyme activities, and hormone contents were analyzed by three-way factorial analysis of variance with cultivar (GT11-80, GT29), fungal strain (wild type, b-locus mutant), and time (1, 3, 5, and 7 dpi) as fixed factors, followed by Tukey’s honestly significant difference test for the comparisons shown in the figures (p < 0.05). Because sampling was destructive, a different set of plants was harvested at each time point, so the design is not a repeated-measures design and a fixed-effects factorial model was used. The complete analysis of variance tables is given in **Supplementary Table 11**. Analyses were performed in GraphPad Prism and R.

## 3 Results

### 3.1 Early quantification of *S. scitamineum* in GT29 and GT11-80 during initial infection

To confirm infection of *S. scitamineum*, we used absolute qPCR to measure bE gene copy number per gram of tissue during the early infection period (1–7 days postinoculation, dpi). In the susceptible cultivar GT11-80, the virulent wild-type (WT) strain grew quickly and steadily, rising from 6.50 × 10^6^ bE gene copies/g at 1 dpi to a peak of 1.72 × 10^7^ bE gene copies/g at 7 dpi. The *b*-locus mutant peaked at 4.52 × 10^6^ bE gene copies/g at 3 dpi and then dropped sharply to 7.50 × 10^5^ copies/g by 7 dpi (**Figure 1A**). In the resistant cultivar GT29, the fungus was inhibited for both strains. WT strain fell constantly from 2.51 × 10^6^ copies/g at 1 dpi to 6.50 × 10^5^ copies/g by 7 dpi, and the mutant showed a similar decline. At 1 dpi, no significant difference was detected between the WT and mutant strains; the mutant was therefore able to enter the host, although these data do not establish that the two strains colonized the host to the same extent. From 3 to 7 dpi, the two strains differed by less than twofold, a range that the difference in bE copy number between them can itself produce (p < 0.05), but the total bE gene copy number was far lower than in the susceptible host (**Figure 1B**). These results show that GT11-80 lets the virulent *S. scitamineum* grow without inhibition, while GT29 actively suppresses colonization soon after inoculation and keeps the bE gene copy number very low.

**Figure 1:**
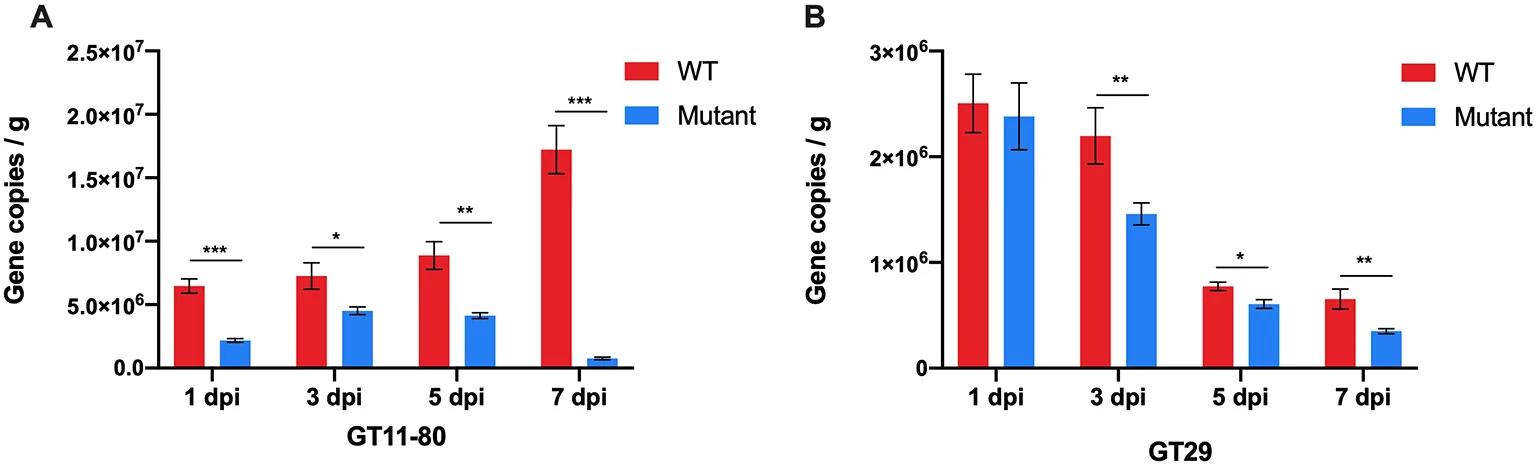
Dynamic quantification of *S. scitamineum* colonization in susceptible and resistant sugarcane cultivars. **(A)** bE gene copy number per gram of tissue in the susceptible cultivar GT11-80 and **(B)** bE gene copy number per gram of tissue in the resistant cultivar GT29 was quantified via absolute qPCR targeting the bE gene at 1, 3, 5, and 7 days postinoculation (dpi). Plants were inoculated with either the wild-type (WT) or the *b*-locus mutant strains. Data are presented as mean ± SD of three biological replicates. Asterisks above the bars indicate statistically significant differences between WT and mutant strains within each time point (*p < 0.05, **p < 0.01, ***p < 0.001), as determined by Student’s *t*-test.

### 3.2 Differential ROS content in early infection

To examine the oxidative response caused by *S. scitamineum* infection, we measured superoxide anion (O_2_^−^) and hydrogen peroxide (H_2_O_2_) during the early infection period (1–7 dpi). Data were analyzed by three-way factorial ANOVA (**Supplementary Table 11**). In superoxide anion (O_2_^−^) dynamics (**Figure 2A**), GT29 produced a stronger and longer oxidative response than GT11-80. Under WT strain infection, O_2_^−^ in GT29 stayed high (group a) from 1 to 5 dpi. The susceptible GT11-80 also started a defense response, but its ROS level was lower than GT29/WT at 1 dpi (group b) and declined by 7 dpi (group b). The *b*-locus mutant gave a weaker response in both cultivars. In GT11-80, O_2_^−^ under the mutant was lower than under the WT at every time point. At 5 dpi, both wild-type-infected groups retained a high O_2_^−^ level (group a), whereas the two mutant-infected groups were significantly lower (group b). This shows that only GT29 kept a high O_2_^−^ level under virulent infection. Hydrogen peroxide (H_2_O_2_) accumulation (**Figure 2B**) followed a similar pattern. GT29 kept higher H_2_O_2_ than GT11-80 through the whole sampling period. At 1 dpi, GT29/WT was highest (group a), GT11-80/WT was intermediate (group ab), and the mutant groups were lower. By 5 dpi, the gap was largest, and GT29/WT was still higher (group a) than all other treatments. At 7 dpi, H_2_O_2_ in GT29 did not differ significantly between WT and mutant infection (both group a), while GT11-80 stayed lower (groups ab and b). In summary, GT29 mounted a faster and more lasting oxidative burst than GT11-80. The lower ROS induction by the *b*-locus mutant was seen in both cultivars and at all time points.

**Figure 2:**
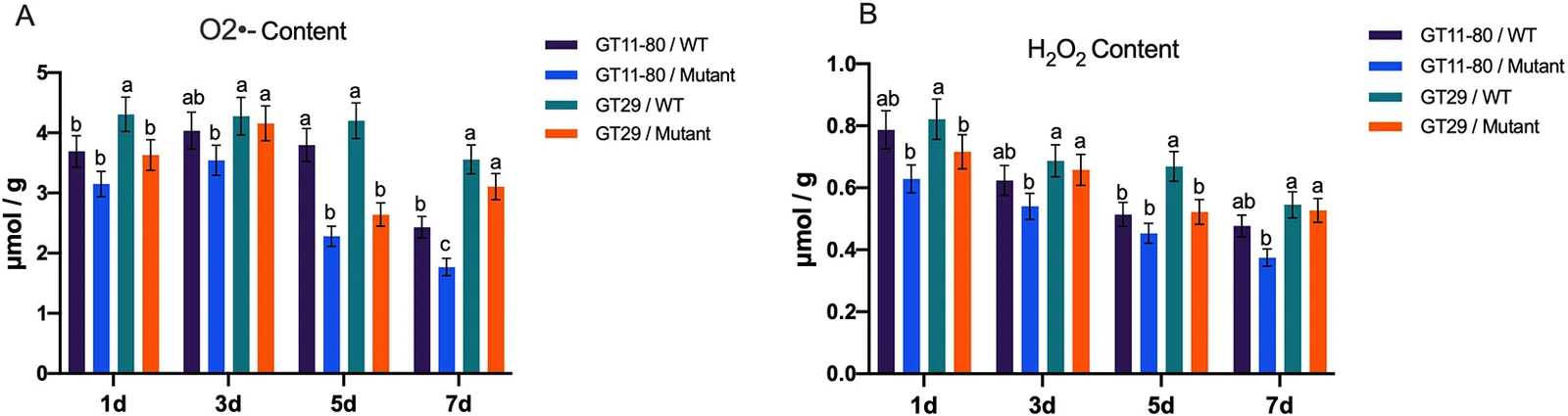
Temporal dynamics of reactive oxygen species (ROS) accumulation in sugarcane basal meristem tissue during early *Sporisorium scitamineum* infection. **(A)** Superoxide anion (O_2_^−^) content and **(B)** hydrogen peroxide (H_2_O_2_) content were quantified in susceptible (GT11-80) and resistant (GT29) sugarcane cultivars. Plants were inoculated with either the wild-type (WT) or the *b*-locus mutant strains, and samples were collected at 1, 3, 5, and 7 days postinoculation (dpi). Data are presented as mean ± SD of three biological replicates. Different lowercase letters above the bars indicate statistically significant differences among treatments within each time point (p < 0.05), as determined by three-way factorial ANOVA followed by Tukey’s honestly significant difference test.

### 3.3 Enzyme activity patterns following smut challenge

We measured seven defense-related enzymes (**Figure 3**). In general, the *b*-locus mutant induced stronger enzyme responses than the WT strain. Hydrolytic enzymes (β-1,3-glucanase and chitinase) showed lower activity to susceptibility. In GT11-80, WT infection repressed β-1,3-glucanase (**Figure 3A**) and chitinase (**Figure 3G**) and kept them at the lowest level (for example, group c). The mutant strain induced higher activity than the wild-type strain in both cultivars, reaching the highest level (group a) in GT29, which suggests that fungal virulence factors repress these enzymes. PAL activity (**Figure 3B**) showed a similar difference. GT29 kept a stable activity, while GT11-80 responded weakly to the WT strain. With the mutant strain, PAL activity in GT11-80 recovered to peak levels (group a) by 7 dpi, which again points to a role of the pathogen in controlling this pathway. ROS-scavenging enzymes (CAT, POD, APX, and SOD): these antioxidants were generally increased to deal with oxidative stress. For CAT (**Figure 3D**), POD (**Figure 3E**), and APX (**Figure 3F**), the mutant strain induced the highest activity, often reaching group a at late infection. GT29 was able to keep these activities high, while GT11-80/WT was often suppressed. SOD (**Figure 3C**) showed the opposite trend and decreased over time. At 7 dpi, WT-infected plants kept higher SOD (group a) than the mutant group (group b), which indicates slower turnover of superoxide signaling under the virulent strain.

**Figure 3:**
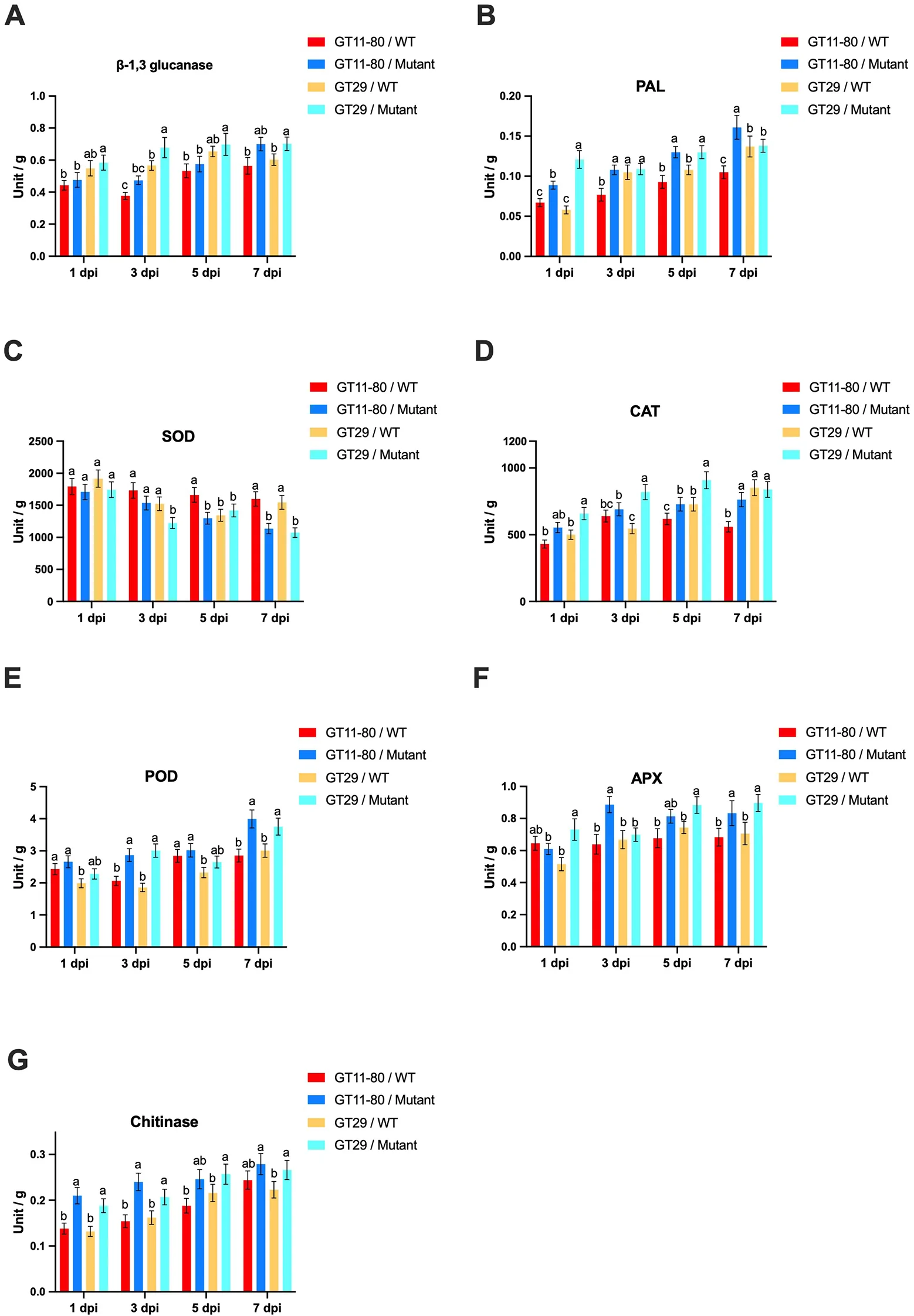
Temporal dynamics of defense-related enzyme activities in susceptible (GT11-80) and resistant (GT29) sugarcane cultivars during early *S. scitamineum* infection. Basal meristem tissue was sampled at 1, 3, 5, and 7 days postinoculation (dpi) following inoculation with either the WT strain or the *b*-locus deletion mutant. The activities of the following enzymes were quantified: **(A)** β-1,3-glucanase, **(B)** phenylalanine ammonia-lyase (PAL), **(C)** superoxide dismutase (SOD), **(D)** catalase (CAT), **(E)** peroxidase (POD), **(F)** ascorbate peroxidase (APX), and **(G)** chitinase. Data represent mean ± SD of three biological replicates (n = 3). Different lowercase letters above the bars indicate statistically significant differences among treatments within each time point (p < 0.05), as determined by three-way factorial ANOVA followed by Tukey’s honestly significant difference test.

### 3.4 Dynamic changes in plant hormones

We measured six phytohormones in the susceptible (GT11-80) and resistant (GT29) cultivars at 1, 3, 5, and 7 dpi (**Figure 4**). Defense-related hormones (JA, ACC (ethylene precursor), SA) showed clear changes after infection. JA (**Figure 4C**) and ACC (**Figure 4F**) rose together and peaked at 5 dpi. The WT strain induced more of these hormones than the *b*-locus mutant. In GT29, WT infection gave the highest JA (group a) and ACC (group a) at 5 dpi. GT11-80 responded more weakly to WT strain infection (group b for both JA and ACC), and the mutant gave the lowest levels (group c or d). SA (**Figure 4E**) showed a gene-for-gene pattern. The mutant strain caused a fast and strong SA increase in GT29, with the highest levels (group a) at 1 and 5 dpi. In GT11-80, SA rose slowly under WT infection and reached its highest level only at 7 dpi, a time point at which the four groups no longer differed significantly, which suggests a delayed and less specific defense. Growth-related hormones (IAA, ABA, and GA) were also affected by the pathogen. For IAA (**Figure 4D**), WT infection kept higher levels than the mutant. GT29 kept high IAA, especially under WT infection at 3 and 7 dpi (group a), while the mutant lowered it (groups c and b, respectively). For ABA (**Figure 4B**), the strains differed mainly at late stages. WT infection kept ABA high (~600–800 ng/g, group a) in both cultivars through 7 dpi, while the mutant caused a sharp drop (~300 ng/g, group b). This indicates that the *b*-locus is needed to keep host ABA high. GA_3_ (**Figure 4A**) increased slowly over time. By 7 dpi, the mutant induced more GA (group a) than the WT strain (group b) in both cultivars. The three-way analysis of variance confirmed a significant effect of the fungal strain on all six hormones, and a significant cultivar × strain × time interaction for SA and IAA, indicating that the b-locus-dependent hormone response followed a different time course in the two cultivars (**Supplementary Table 11**). In summary, GT29 showed an earlier and stronger hormone response, with high JA and ACC at 5 dpi and a fast SA increase against the mutant strain.

**Figure 4:**
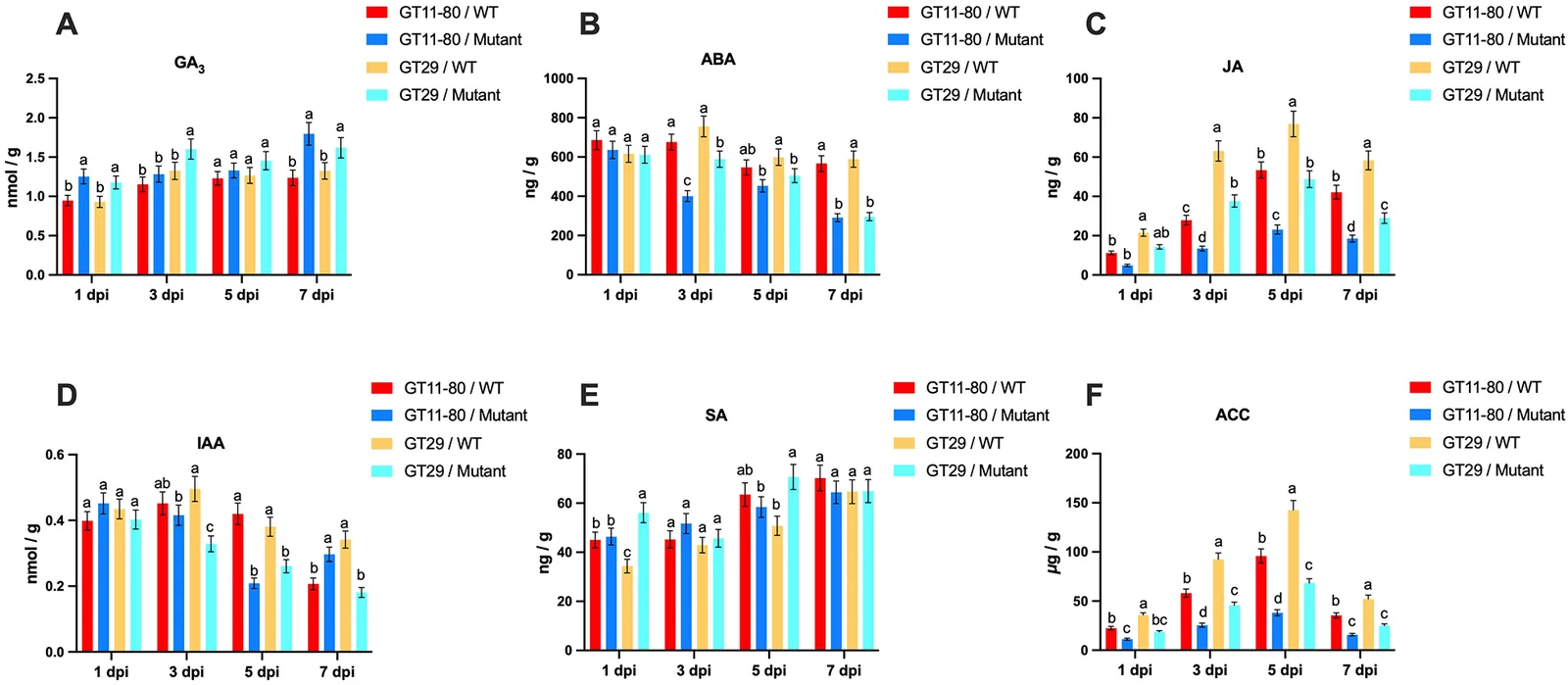
Dynamics of endogenous phytohormone levels in susceptible (GT11-80) and resistant (GT29) sugarcane cultivars during early *Sporisorium scitamineum* infection. Plants were inoculated with either the WT strain or the *b*-locus deletion mutant, and basal meristem samples were collected at 1, 3, 5, and 7 days postinoculation (dpi). The concentrations of the following hormones were quantified: **(A)** gibberellic acid (GA_3_), **(B)** abscisic acid (ABA), **(C)** jasmonic acid (JA), **(D)** indole-3-acetic acid (IAA), **(E)** salicylic acid (SA), and **(F)** 1-aminocyclopropane-1-carboxylic acid (ACC). Data are presented as mean ± SD of three biological replicates (n = 3). Different lowercase letters above the bars indicate statistically significant differences among the four treatment groups within each time point (p < 0.05), as determined by three-way factorial ANOVA followed by Tukey’s honestly significant difference test. Hormone contents are expressed as nmol/g for GA3 and IAA, ng/g for ABA, JA, and SA, and µg/g for ACC, in accordance with the units of the corresponding calibration standards (**Supplementary Table 9**).

### 3.5 Time-ordered gene co-expression networks reveal *b*-locus-dependent transcriptional cascades in the susceptible variety

To study the transcriptional program that depends on the fungal *b*-locus, we built a TO-GCN in the susceptible variety GT11-80 from transcription factors (TFs) that were positively co-expressed under WT infection (W^+^) but not correlated under mutant infection (M0). The network describes co-expression relationships and their temporal order. We used an ethylene-responsive factor (*EREBP*, SoffiXsponR570.02Dg300000) as the seed, because it showed the highest expression among the early-peaking EREBP family members at 0 dpi (FPKM = 30.78 in GT11-80 and 8.11 in GT29 under WT infection) and has well-documented roles in plant immunity. This gave 2,539 TFs forming 71,964 W^+^-specific TF–TF co-expression pairs (**Supplementary Table 1**). The expression profile of the seed gene across all five time points is shown in **Supplementary Figure 1**. With a breadth-first search (BFS), the TFs were placed into 14 ordered levels (L1–L14) in the W^+^ TO-GCN (**Figure 5A**), from early to late modules. The largest modules were at L2 (220 TFs) and L3 (518 TFs), and the modules became smaller down to L14 (2 TFs). This points to a strong early regulatory wave followed by finer downstream regulation.

**Figure 5:**
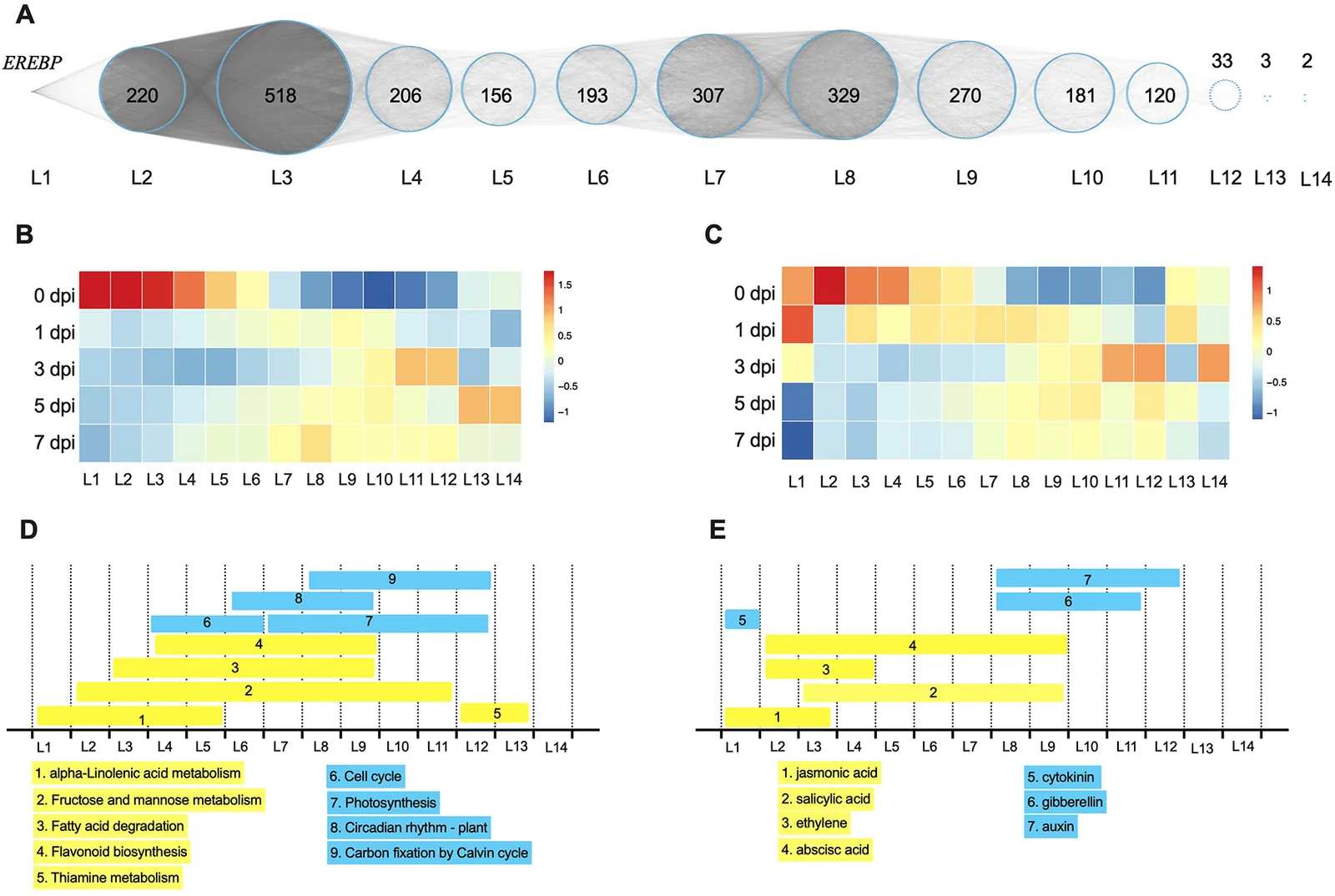
TO-GCN reveals *b*-locus-dependent transcriptional cascades in the susceptible sugarcane cultivar (GT11-80). **(A)** Topology of the TO-GCN constructed using the *EREBP* transcription factor (SoffiXsponR570.02Dg300000) as the seed node. The network comprises TFs that are positively co-expressed under wild-type (W^+^) infection but uncorrelated under mutant (M0) infection. Nodes were organized into 14 hierarchical levels (L1–L14) using a breadth-first search (BFS) algorithm. The circle size and numbers indicate the count of TFs assigned to each level. **(B, C)** Temporal expression heatmaps of the mapped TFs across the 14 levels under **(B)** W^+^ infection and **(C)** M0 infection. The color scale represents row-normalized z-scores of FPKM values across 0, 1, 3, 5, and 7 dpi. A clear diagonal shift of peak expression from early (L1–L3) to late (L8–L14) levels is observed in W^+^ but is disrupted in M0. **(D)** KEGG pathway enrichment analysis of genes co-expressed with TFs at each level. Colored bars span the levels where specific metabolic and cellular processes are significantly enriched. **(E)** Enrichment of hormone-related GO terms across network levels. Yellow bars indicate defense-associated hormones (e.g., jasmonic acid, salicylic acid, and ethylene) enriched in early levels, while blue bars indicate growth-associated hormones (e.g., gibberellin and auxin) enriched in late levels.

The order of the levels was set by TF expression over the five time points (0, 1, 3, 5, and 7 dpi), using z-score normalized FPKM values. Under W^+^ (**Figure 5B**), the expression showed a diagonal pattern: low levels (L1–L3) peaked early (0–1 dpi), mid-levels (L4–L7) peaked at 3 dpi, and high levels (L8–L12) peaked at 5–7 dpi. This steady shift over time fits the BFS level assignment and suggests that regulation is passed from one level to the next. Under M0 (**Figure 5C**), this diagonal pattern was mostly gone, and the TF levels did not show clear peaks at separate time points. So without the *b*-locus, no clear time-ordered cascade was formed. These results indicate that the wild-type *b*-locus is required for the ordered TF program in the susceptible genotype, starting with early ethylene-responsive factors and moving through defined levels. This *b*-locus-dependent cascade is lost under mutant infection, which shows that the *b*-locus helps coordinate host transcription during early infection.

### 3.6 Functional analysis of *b*-locus-dependent genes in the susceptible variety

In GT11-80 (W^+^ genotype), KEGG enrichment of the *b*-locus-dependent co-expression genes showed stepwise activation across levels 1 to 14, mainly for defense structures and metabolic changes (**Figure 5D**; **Supplementary Table 2**). The middle levels (levels 6–7) were enriched for plant cell wall organization, which suggests that the plant tried to strengthen physical barriers against the fungus. Because this happened only in the mid-early levels, the attempt to block hyphal penetration was probably not efficient. No ROS biosynthetic process was enriched, which suggests weak defense against this biotrophic pathogen. Phenylpropanoid biosynthesis appeared later (levels 7–9) and points to the production of secondary metabolites such as lignin and phenolics; their late timing may help the pathogen become established before defenses are fully active. Metabolic pathways were activated from mid to late stages: photosynthesis (levels 7–12) and carbon fixation by the Calvin cycle (levels 8–12) suggest a reactive shift to keep up energy under stress, and circadian rhythm—plant (levels 3–12) enrichment suggests changes in immune timing that the pathogen may use to its advantage. In GT11-80, then, the response was spread out and loosely organized, with early structural defenses followed by delayed metabolic and oxidative changes.

In the susceptible genotype under W^+^ infection, the hormone-related GO terms showed a staged but weak signaling pattern (**Figure 5E**; **Supplementary Table 3**). Jasmonic acid (L1–3), cytokinin (L1), and ethylene (L2–4) were activated early, in line with a quick start of basal defense. The activation windows were narrow, though, which suggests that this early signaling was brief or weak. Abscisic acid and salicylic acid stayed enriched from L2 to L9, which suggests a long stress response. SA is usually linked to resistance against biotrophic pathogens, but its partial overlap with ABA, which can suppress defense, suggests an unbalanced hormone state that the pathogen may use. At later stages (L8–11), auxin and gibberellin terms appeared and point to a shift toward growth. This is consistent with a shift in host development that is associated with susceptibility. In short, the *b*-locus allowed the hormones to be activated in sequence, but the timing favored infection rather than effective resistance.

### 3.7 TO-GCN analysis in the resistant variety

Using the same W^+^/M0 criterion as for GT11-80, we built a TO-GCN in the resistant variety GT29 from TF–TF coexpression pairs that were positive under wild-type (W^+^) infection and not correlated under mutant (M0) infection. This network is therefore defined by the same *b*-locus-dependent criterion as the susceptible network; what differs between the two cultivars is how the expression of the selected transcription factors behaves under the two infection conditions. We found 2,380 transcription factors forming 31,977 W+/M0 coexpression pairs. These were placed into 12 time-ordered levels (L1–L12) by breadth-first search (BFS), forming one time-ordered cascade (**Figure 6A**). In this network, most TFs were concentrated at L4–L9, and the largest modules were at L4 (437 TFs) and L5 (422 TFs). This points to a strong regulatory phase in the mid-early to mid-late windows (**Supplementary Table 4**). Levels L1–L3 and L10–L12 had fewer TFs, so early and late activity was more limited. In the susceptible W^+^ network, by contrast, the TFs peaked earlier (L2–L3), which fits a stronger immediate response under *b*-locus activation. The expression heatmaps over the time points supported the level assignments. Under both W^+^ and M0 (**Figures 6B, C**), TF expression followed a smooth progression in time: L1–L3 were mainly active at 0–1 dpi, L4–L6 at 1–3 dpi, and L7–L9 peaked at 3–5 dpi. L10–L12 peaked later, at 5–7 dpi. In the resistant host, both conditions showed a similar temporal expression pattern (clear diagonal in z-score heatmaps), so the TF programs ran in order whether or not the *b*-locus was present. In the susceptible genotype, deleting the *b*-locus broke the cascade and the expression lost its order over time. These results suggest that in the resistant genotype, the host TF program depends less on pathogen *b*-locus signals to start and run its transcriptional cascades. The temporal expression pattern was similar under W^+^ and M0, which points to a more stable, preset transcriptional framework in resistant sugarcane. This stability may help the plant limit fungal interference and slow disease.

**Figure 6:**
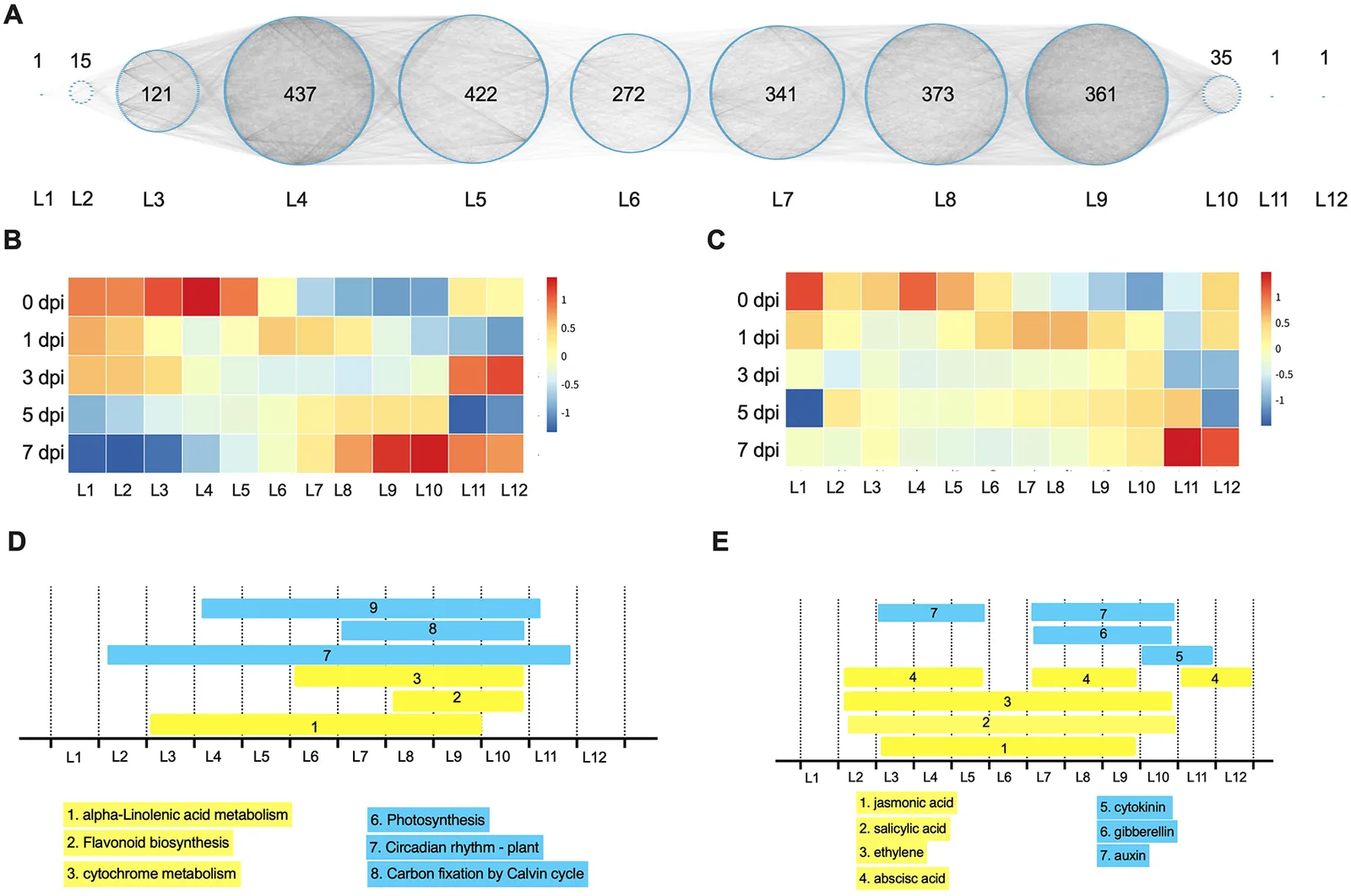
Characterization of *b*-locus-associated transcriptional cascades in the resistant sugarcane cultivar (GT29) via TO-GCN. **(A)** Topology of the TO-GCN constructed from transcription factors (TFs) that exhibit positive coexpression under wild-type (W^+^) infection but lack correlation under mutant (M0) infection. TFs were organized into 12 hierarchical levels (L1–L12) using a BFS strategy. The circle size corresponds to the number of TFs, highlighting a centralized distribution with major regulatory modules peaking at mid-stages (L4–L5). **(B, C)** Temporal expression heatmaps of the mapped TFs across the 12 levels under **(B)** W^+^ infection and **(C)** M0 infection. The color scale represents row-normalized z-scores of FPKM values across 0, 1, 3, 5, and 7 dpi. A coherent diagonal shift of peak expression from early (L1–L3) to late (L10–L12) levels is observed in both W^+^ and M0 conditions, that is, the temporal order of these transcription factors is retained in the resistant cultivar under both conditions. **(B, C)** show the expression of the same set of W^+^/M0 network nodes projected onto the two infection conditions; no separate network was constructed from the mutant data. **(D)** Functional enrichment analysis of genes co-expressed with TFs at each level. Bars indicate the specific levels enriched for key metabolic and structural defense processes. **(E)** Enrichment of hormone-related GO terms across network levels. Yellow bars represent defense-associated hormones showing broad and sustained activation, while blue bars represent growth-regulating hormones enriched in mid-to-late levels.

### 3.8 Functional analysis of *b*-locus-dependent genes in the resistant variety

In the resistant variety GT29, enrichment of *b*-locus-dependent genes across levels 1 to 12 showed a more coordinated activation of defense and metabolic pathways (**Figure 6D**; **Supplementary Table 5**). Plant cell wall organization was enriched early (levels 2–5), which suggests fast strengthening of cell barriers to block fungal entry. ROS biosynthetic process followed (levels 8–10), which suggests a controlled oxidative response that produces signaling molecules and has antimicrobial effects without too much tissue damage. Phenylpropanoid biosynthesis appeared at mid stages (levels 7–9) and supports timely production of defense compounds such as flavonoids and lignins. Metabolic pathways were enriched earlier and over a wider range: photosynthesis (levels 2–11) and carbon fixation by the Calvin cycle (levels 4–10) suggest active energy management to support defense. Circadian rhythm—plant (levels 6–10) overlapped widely and may help time the expression of immune genes. In GT29, then, early structural and oxidative defenses were combined with steady metabolic support.

In the resistant genotype, the hormone-related GO terms show a coordinated defense, with several signaling pathways turned on in a controlled way (**Figure 6E**; **Supplementary Table 6**). Jasmonic acid (L3–9), a main regulator of defense against necrotrophs and wounding, stayed active over a long period, which suggests steady local defense. Ethylene signaling (L2–9), which often acts together with JA, supports fast immune responses, especially cell wall strengthening and antimicrobial gene expression. The long co-enrichment of JA and ET suggests an early and lasting front-line defense. Abscisic acid (L3–11), usually linked to abiotic stress, was enriched over a wide range and may play a dual role: setting defense thresholds and keeping the immune response from becoming too strong and damaging the plant. Its late activity may also help the cells return to normal after the immune response. Salicylic acid (L2–10), a key hormone in defense against biotrophic pathogens, was strongly and steadily enriched. This supports a strong, pathogen-specific defense that started early and lasted through the infection. In the susceptible genotype, SA appeared later, which may reflect a slower defense. Cytokinin (L9–10) was enriched at later levels and may help immune cells divide or keep metabolism going under stress. Gibberellin (L8–10) and auxin (L3–5, L8–10) are usually linked to growth, but they were also enriched here. In resistant plants, the short activation of these growth hormones may help repair tissue or strengthen barriers after infection, rather than being used by the pathogen as in the susceptible genotype. In short, the resistant genotype turned on defense and growth hormones over a wider time range and with more overlap. This suggests a flexible, layered defense that combines a strong early pathogen-specific response (JA and ET) with longer-term balance and development (ABA, auxin, GA), to limit the fungus and protect the tissue.

### 3.9 JA/ET-mediated defense responses to the *b*-locus

To study the JA/ET-mediated defense responses, we focused on these genes. We took *CTR1*, *EIN2*, *EIN3*, *JAZ*, *COL1*, *ERF1*, and *WRKY33* from the full TO-GCN in the susceptible and resistant varieties. The interaction topology of *EIN3*, *JAZ*, *ERF1*, and *WRKY33* differed clearly between the susceptible variety GT11-80 and the resistant variety GT29 (**Figure 7**). In GT11-80, *ERF1* (SoffiXsponR570.01Cg002500) is in a central position and is broadly correlated with many *JAZ* genes, as well as with *WRKY33* and *EIN3*. This suggests that in the susceptible background, *ERF1* acts as a main hub for ethylene and jasmonate signaling, which fits its known role as a downstream target of *EIN3*/*EIL1* and a co-regulator with *WRKY33*. Such overactivation of JA/ET-mediated defense can, in some cases, especially under biotrophic or hemibiotrophic attack, help the disease progress. In the resistant variety GT29, *ERF1* is no longer among the highly connected nodes, its degree falling from 352 in GT11-80 to 20 in GT29, and one *EIN3* homolog (SoffiXsponR570.01Bg347100) is correlated with many *JAZ* genes and a defined group of *WRKY33* members. This change suggests that GT29 relies more on *EIN3*-mediated signaling than on *ERF1*.

**Figure 7:**
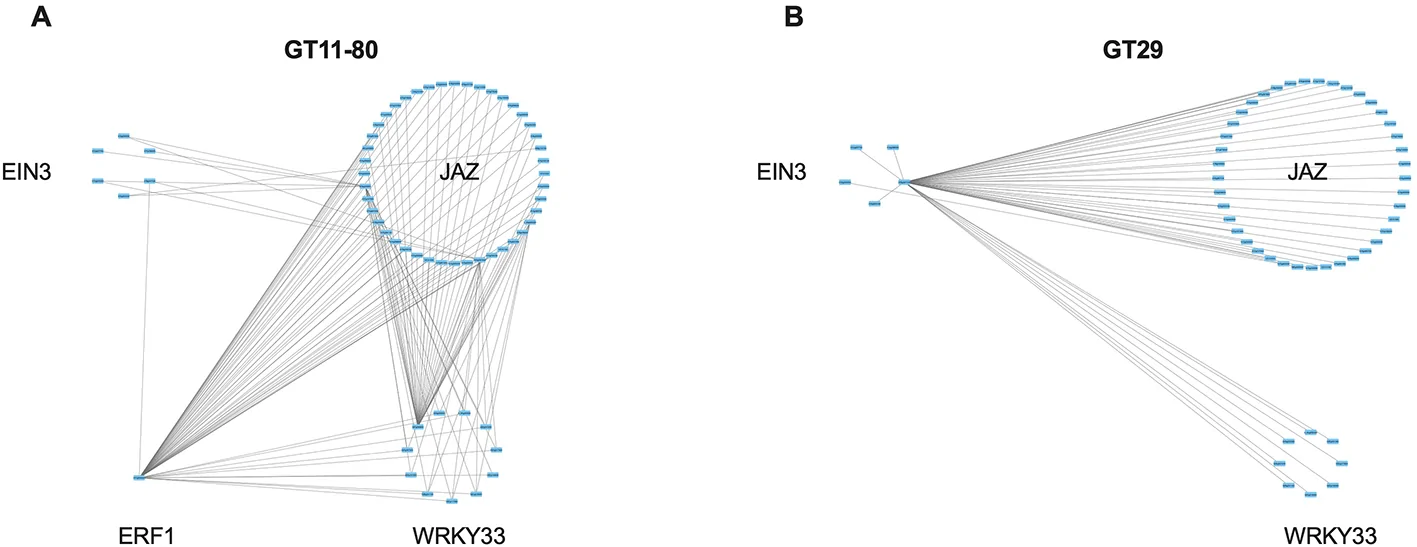
Distinct co-expression topologies of JA/ET signaling components in susceptible (GT11-80) and resistant (GT29) sugarcane cultivars. Subnetworks were extracted from the global TO-GCN to highlight interaction patterns among key JA/ET pathway genes, including *EIN3*, *JAZ*, *ERF1*, and *WRKY33*. **(A)** The interaction network in the susceptible cultivar GT11-80. *ERF1* (SoffiXsponR570.01Cg002500) occupies a central hub position, exhibiting broad positive correlations with multiple *JAZ*, *WRKY33*, and *EIN3* genes. This suggests an *ERF1*-dominant integration of JA/ET signaling in the susceptible background. **(B)** Interaction network in the resistant cultivar GT29. In contrast to GT11-80, *ERF1* is not connected to the JA/ET module shown here. Instead, a specific *EIN3* homolog (SoffiXsponR570.01Bg347100) acts as the central hub, displaying extensive correlations with a large cluster of *JAZ* genes and *WRKY33* members. This shift indicates a preference for *EIN3*-mediated signaling branches over *ERF1*-driven outputs in the resistant cultivar. Hub status was assessed quantitatively rather than visually. In GT11-80, ERF1 ranks in the top 2% of the network by degree (352 connections, rank 43 of 2,539) and in the top 8% by closeness, whereas in GT29, it falls to the 55th percentile (20 connections). EIN3 shows the opposite pattern, rising from the 83rd percentile in GT11-80 to the top 0.3% by degree, the top 0.7% by betweenness and the top 0.6% by closeness in GT29. Because the two networks differ in density, these comparisons are based on ranks within each network. The full values are given in **Supplementary Table 12**.

## 4 Discussion

The mating-type *b*-locus of *Sporisorium scitamineum* encodes a heterodimeric transcription factor (bE/bW) that is required for fungal filamentation and pathogenic development (Yan et al., 2016). Deleting the *b*-locus removes mating and virulence in the fungus, which shows that it is a key virulence factor. In this study, we used a *b*-locus mutant of *S. scitamineum* to test how fungal virulence factors shape host defense. We compared a resistant cultivar (GT29) and a susceptible cultivar (GT11-80) after infection with the wild-type or the *b*-mutant strain. This let us examine the transcriptional and physiological changes caused by a fully virulent pathogen and by a virulence-impaired strain.

Time-ordered gene co-expression network (TO-GCN) analysis is a time-series method that builds a co-expression network of transcription factors ordered by when they are expressed (Chang et al., 2019). In practice, picking a starting TF with a clear time trend and using breadth-first search to link co-expressed TFs into successive levels map the order of gene activation over time. Because it uses time information, this method can show dynamic regulatory patterns that static analyses miss (Wong et al., 2020). TO-GCN has been useful in plant pathogen studies. Li et al. applied it to resistant and susceptible grapevine infected with white rot and found that the resistant cultivar turned on defense TFs quickly in early infection (Li et al., 2024). Wu et al. studied sugarcane infected with the smut fungus (*Sporisorium scitamineum*) and used TO-GCN to sort 2,459 differentially expressed genes (DEGs) into 10 time-ordered levels. They found stress-related modules, such as Ca²^+^ signaling pathways, in the defense response (Wu et al., 2024). In our study of *b*-locus-dependent virulence, TO-GCN let us build *b*-locus-dependent transcriptional networks and find genotype-specific TF cascades. This showed how the *b*-locus changes the timing of defense gene activation and produces the resistance differences we observed.

We compared the TO-GCN structures of the susceptible cultivar GT11-80 and the resistant GT29 and found different regulatory strategies that may explain their different defense outcomes. In GT11-80, the *b*-locus-dependent network had 2,539 TFs and 71,964 coexpression edges across 14 layers, and most TFs were clustered in the early layers L2 and L3 (**Figure 5A**). This points to a fast, early transcriptional response that was broad but not well organized. The GT29 network had 2,380 TFs and 31,977 edges across 12 levels, with TFs concentrated in the mid-levels L4 and L5 and spread more evenly over time (**Figure 6A**). This points to a more balanced, modular response. Similar mid-layer clustering has been seen in *Arabidopsis* and rice resistance networks, where stable defense comes from distributed, time-staged transcriptional control (Zhang et al., 2017). The early TF surge in GT11-80 may be a strong but poorly coordinated early defense response, which fits other susceptible interactions where early responses that are mis-timed or too strong weaken defense (Fung et al., 2008; Maggiolini et al., 2025). GT29 seems to mount a steadier defense that may rely on preset regulatory modules less open to pathogen interference. This view fits the smoother activation in the GT29 heatmaps and the more disrupted pattern in GT11-80. These differences show that TO-GCN can reveal the timing of regulation that static networks miss. By keeping the time order, TO-GCN shows when specific TF modules are turned on, which helps identify key points in disease resistance (Chang et al., 2019; Li et al., 2024). Our results suggest that the resistant genotype runs a more efficient, well-timed transcriptional cascade, while the susceptible genotype responds early and in a less organized way.

To understand how the TF genes relate to the genes correlated with them, we analyzed the function of the correlated genes level by level. Comparing the KEGG and GO enrichment from the *b*-locus-dependent coexpression modules in the susceptible and resistant varieties showed clear differences in the timing of defense, metabolic, and hormonal responses. These differences show that the *b*-locus is required for the observed timing of pathway activation, whereas the outcome, resistance or susceptibility, is associated with the host genotype. Comparing the enrichment profiles of the two varieties shows that the *b*-locus-dependent responses differ between the two hosts over time. In GT29, the pathways were activated earlier and overlapped more (**Figure 6D**), while in GT11-80, they were later and more fragmented (**Figure 5D**). In the resistant variety, plant cell wall organization started at level 2 and ran to level 5, which allowed fast barrier strengthening to limit *S. scitamineum* hyphae. This fits studies showing that cell wall glycoproteins and silicon-based reinforcement are linked to less smut colonization. In GT11-80, the same pathway covered only about two levels in the middle stage, which may let the pathogen enter before defenses are fully active. The ROS biosynthetic process supports the oxidative bursts that limit biotrophic fungi. It was enriched earlier and longer (levels 8–10) in the resistant variety but not in the susceptible one, which matches the higher ROS enzyme activity in resistant cultivars that limits fungal spread through balanced production and scavenging. The lack of an ROS response in susceptible plants may mean weak ROS signaling and higher vulnerability. Metabolic reprogramming also differed: photosynthesis and carbon fixation by the Calvin cycle started sooner and lasted longer in the resistant variety (levels 2–11 and 4–10) than in the susceptible one (levels 7–12 and 8–12). This may reduce the infection-related drop in photosynthesis and prevent the sink–source imbalance that helps the fungus get nutrients in susceptible hosts. These results suggest that the fast, well-timed response in GT29 saves resources and supports the energy needs of a long immune response without harming the plant, while the delays in GT11-80 help the disease progress (He et al., 2024; Cheaib and Killiny, 2025). These patterns fit earlier work on sugarcane smut caused by *S. scitamineum*, in which resistant cultivars keep up photosynthesis to support defense, while susceptible ones have reduced carbon assimilation and lose yield. Our previous studies showed that pathogen-induced metabolic changes in susceptible sugarcane often cause sink–source imbalances that help the fungus take up nutrients (Song et al., 2022). Combining the physiological data with the TO-GCN links these mechanisms. For example, the strong ROS bursts and enzyme activity in GT29 (**Figures 2**, **3**) match the steady detoxification enrichment (levels 6–10, **Figure 6D**), which supports toxin removal and energy rebalancing through photosynthesis. The comparison between the two strains should, however, be read with care. Reactive oxygen species accumulation was consistently lower under mutant infection, which is compatible with a contribution of b-locus-dependent fungal activity to the host oxidative response; because the two strains did not necessarily present the host with the same amount of fungal stimulus, an effect of the amount of colonization cannot be excluded on the basis of the present data.

For the hormone-related GO terms, the susceptible variety showed a staged but weak signaling pattern. JA (levels 1–3), cytokinin (level 1), and ethylene (ET, levels 2–4) were activated early and briefly, followed by longer activation of abscisic acid (ABA) and salicylic acid (SA) (levels 2–9). The narrow early windows suggest weak basal defense. The overlap of ABA and SA may create an antagonistic state that the pathogen can use, because ABA often suppresses SA-based resistance to biotrophs (Jiang et al., 2010; Häffner et al., 2015). These inferences are supported by the measured hormone levels (**Figure 4**). Under wild-type infection, JA (**Figure 4C**) and ACC (**Figure 4F**) in GT11-80 reached only group b at 5 dpi, below the levels in GT29. SA (**Figure 4E**) rose slowly and reached its highest level only at 7 dpi, while ABA (**Figure 4B**) stayed high (~600–800 ng/g). High ABA together with late SA fits the ABA–SA antagonism described above and points to a slow, poorly timed defense. This unbalanced state may help the fungus, as shown by the late auxin and gibberellin (GA) enrichment (levels 8–11). These changes likely alter host growth in ways that favor infection, such as teliospore spread (Ma and Ma, 2016). Other work on plant–pathogen interactions shows that biotrophic fungi, including smut pathogens, use auxin signaling to cause galls or overgrowth, which increases susceptibility in hosts without well-coordinated defenses (Nagarajan et al., 2023).

In the resistant variety, the hormones were enriched over a wider range, with more overlap and longer activation than in the susceptible variety (**Figure 6E**). Jasmonic acid (JA; levels 3–9) and ethylene (ET; levels 2–9) were activated together over a long period, which strengthens immunity through antimicrobial gene expression and the hypersensitive response. The measured hormones support this pattern (**Figure 4**). Under wild-type infection, GT29 reached the highest JA (**Figure 4C**) and ACC (**Figure 4F**) levels at 5 dpi (group a), clearly above GT11-80. Because ACC is the direct precursor of ethylene, its accumulation is consistent with an engagement of the ethylene branch, which agrees with the broad, sustained JA/ET enrichment in the resistant network, although ethylene itself was not measured. Salicylic acid (SA) was enriched strongly and early (levels 2–10), which is consistent with an early activation of SA-dependent defense. This is an important difference from the delayed SA response in the susceptible variety, and early SA signaling has been associated with restriction of biotrophic pathogens (Chen et al., 2023). This early SA increase fits earlier work showing higher SA in smut-resistant sugarcane cultivars, which is linked to fewer symptoms and stronger defense. The long activation of abscisic acid (ABA) (levels 3–11) likely helps set defense thresholds, preventing autoimmunity without strongly antagonizing the other hormones (García-Andrade et al., 2020). The late enrichment of cytokinin (levels 9–10), gibberellin (GA; levels 8–10), and auxin (levels 3–5, 8–10) suggests controlled growth regulation that helps repair tissue after infection and may counter the pathogen’s effect on host development. This layered hormone strategy is similar to what is seen in model plants such as *Arabidopsis*, where JA/ET–SA crosstalk, although partly antagonistic, supports broad-spectrum resistance through connected signaling networks (Zhou et al., 2022; Proietti et al., 2013). In sugarcane, resistant hybrids have higher SA levels that are directly linked to fewer whip smut symptoms, which supports the timing patterns we found here.

The JA/ET signaling components showed clearly different topologies in the susceptible GT11-80 and the resistant GT29, which gives insight into how the *b*-locus of *Sporisorium scitamineum* is associated with genotype-specific defense. In GT11-80, *ERF1* is in a central hub position, with broad positive correlations to *JAZ* genes, *WRKY33*, and *EIN3*, so it acts as a main integrator of the ethylene (ET) and jasmonic acid (JA) pathways. This may produce a fast but poorly coordinated response. *ERF1* activation, which co-regulates with *WRKY33* in antimicrobial gene expression, could cause problems such as antagonism with salicylic acid (SA) signaling, and this can help a biotrophic pathogen like smut progress (Li et al., 2019). Studies in model plants such as *Arabidopsis* show that *ERF1*, a target of *EIN3*/*EIL1*, increases JA/ET synergy for defense against necrotrophs, but it can weaken resistance to biotrophs if it is overexpressed or misregulated (Berrocal-Lobo et al., 2002). This fits the higher susceptibility of smut-sensitive sugarcane, where hormone imbalance favors fungal colonization (Huang et al., 2015; Zhou et al., 2022). In GT29, *ERF1* is much less connected, and *EIN3* is broadly correlated with *JAZ* and a discrete set of *WRKY33* modules, which points to a shift toward *EIN3*-centric signaling. This shift may improve efficiency: *EIN3* directly derepresses JA/ET-responsive genes, avoids *ERF1*-mediated branches that pathogen effectors might target, and brings in *WRKY33* for specific defense outputs. *EIN3* is the central transcription factor of ethylene signaling, so the high ACC measured in GT29 (**Figure 4F**) is compatible with, but does not demonstrate, an active *EIN3*-centered ethylene branch in the resistant variety. It should be noted that ethylene emission and ethylene-responsive marker genes were not measured in this study, so the involvement of the ethylene branch is inferred only from ACC, the direct precursor of ethylene, together with the co-expression of ethylene-related transcription factors. The weaker JA and ACC accumulation in GT11-80 is consistent with the less effective, *ERF1*-centered output inferred for the susceptible variety. This fits earlier work showing that *EIN3*/*EIL1* act as *JAZ*-associated hubs that coordinate JA and ET for broad-spectrum immunity (Zhu et al., 2011) and that *WRKY33* strengthens responses against fungal pathogens such as *Botrytis cinerea*, a necrotroph, while remaining adaptable in biotrophic settings through network modularity (Meng et al., 2013). Consistent with our observation that WRKY-containing modules differ between the two genetic backgrounds, a recent study showed that *ScWRKY2* was downregulated in smut-resistant but upregulated in smut-susceptible sugarcane cultivars and that its overexpression compromised resistance to *S. scitamineum* (Wang et al., 2025). This supports the view that the cultivar-specific wiring of WRKY-associated modules, rather than the mere presence of these transcription factors, is associated with the contrasting resistance outcomes. In sugarcane, resistant cultivars often show such well-organized hormone networks and can switch defense pathways depending on the virulence of the smut isolate, which lowers teliospore production and whip symptoms compared with susceptible cultivars (Agisha et al., 2022). The defense signaling networks in the two genotypes differed in structure. In the susceptible genotype, the JA/ET signaling pathway adopts an *ERF1*-centric regulation. *ERF1* is the main hub and is broadly and strongly correlated with downstream genes such as *JAZ*, *WRKY33*, and *EIN3*. This highly connected structure may give a non-specific or excessive immune response that can be redirected toward pathways favoring susceptibility or toward metabolic demands that the pathogen uses, which fits the weak and ineffective early defense noted above. In the resistant genotype, the network has an *EIN3*-centric defense topology, in which *ERF1* is separated from the core network. Here, signaling depends mainly on the upstream regulator *EIN3*, which is co-expressed with *JAZ* members and specific *WRKY33* genes. This structure may give the resistant host more direct control and support the lasting JA/ET/SA response needed for effective resistance, avoiding the signaling problems seen in the susceptible variety.

Several limitations of the present study should be noted. Inoculation was carried out by injection at the basal meristem, which causes mechanical wounding and bypasses part of the natural infection barriers. Although the mock treatment received the same injection procedure and the same volume of sterile water, and the wounding was therefore identical in all treatments, the mock samples were used only as a procedural control during inoculation and were not analyzed. Consequently, the wound-induced component of the host response cannot be separated from the infection-induced component, and the reactive oxygen species, defense enzyme, hormone, and transcriptome data reported here should be interpreted as the difference between the WT strain and the b-locus mutant strain within each cultivar, rather than as the total effect of infection relative to a non-inoculated baseline. Our conclusions therefore concern the b-locus-dependent component of the host response, and a wound-only control analyzed in parallel would be required to fully resolve the wounding effect in future work. A further limitation concerns the co-expression network itself. Although the correlations were computed across 15 libraries per condition, these libraries represent only five distinct time points, so the correlation coefficient of any individual TF–TF pair is estimated with limited precision and no single edge should be overinterpreted. Accordingly, we did not draw conclusions from individual edges, and the feature that we interpret is the temporal order of the transcription factor levels, which was robust both to the choice of the seed node and to the choice of the correlation cutoff (**Supplementary Figures 2**, **5**). The TO-GCN presented here should therefore be regarded as an exploratory, hypothesis-generating framework rather than as a validated regulatory network.

## 5 Conclusion

In this study, we used TO-GCN analysis together with network topology to study how sugarcane defense responds to *S. scitamineum* infection. By identifying the transcription factors (TFs) expressed at different infection stages, we built a framework of the transcriptional changes over time in both the susceptible and the resistant background. The topology analysis showed that the result of the JA/ET signaling pathway depends on how the core transcription factors are connected. In the susceptible genotype, the network centers on an *ERF1*-centric signaling hub, which is associated with a less coordinated signaling pattern and with disease progression. In the resistant genotype, the network keeps a coordinated defense by shifting to an *EIN3*-centric regulation, which gives a more efficient and longer-lasting activation of defense pathways. The metabolic and hormonal enrichments, including steady detoxification and photosynthesis rebalancing in the resistant variety, give further support for these differences. From this combined analysis, we propose a working model, which requires functional validation, of how the *b*-locus signal is handled differently in the resistant and the susceptible host. In summary, comparing infection by a wild-type strain and a b-locus deletion mutant in a resistant and susceptible sugarcane cultivar showed that the topology of the JA/ET network differs between the two cultivars, which points to network structure as a factor in sugarcane resistance to smut.

## Data Availability

The datasets presented in this study can be found in online repositories. The raw RNA-seq reads of the 60 libraries have been deposited in the NCBI Sequence Read Archive under BioProject accession number PRJNA1489401 (https://www.ncbi.nlm.nih.gov/bioproject/PRJNA1489401). The transcription factor co-expression edge lists, the GO and KEGG enrichment results, the TO-GCN level assignments, the expression matrix of the network transcription factors and the analysis scripts are provided in the Supplementary Material (**Supplementary Tables 1**–**6**, **13** and **14**, and **Supplementary Data Sheet 1**).

## Supplementary material

supplementary Table S1 Transcription factor co-expression pairs used to construct the susceptible GT11-80 TO-GCN.

supplementary Table S2 KEGG enrichment results for b-locus-dependent genes across GT11-80 TO-GCN levels.

supplementary Table S3 GO enrichment results for b-locus-dependent genes across GT11-80 TO-GCN levels.

supplementary Table S4 Transcription factor co-expression pairs used to construct the resistant GT29 TO-GCN.

supplementary Table S5 KEGG enrichment results for b-locus-dependent genes across GT29 TO-GCN levels.

supplementary Table S6 GO enrichment results for b-locus-dependent genes across GT29 TO-GCN levels

supplementary Table S7 Primer sequences used for SYBR Green qPCR quantification of S. scitamineum bE gene.

supplementary Table S8 Quality-control and mapping statistics of the 60 RNA-seq libraries, including raw and clean read numbers, Q20, Q30, GC content and mapping rates.

supplementary Table S9 Validation parameters of the LC-MS/MS method used for phytohormone quantification, including MRM transitions, calibration ranges, limits of detection and quantification, recoveries, and the lowest measured group mean relative to the limit of quantification for each analyte.

supplementary Table S10 Reaction principle, detection wavelength and definition of one unit of enzyme activity for each of the seven commercial assay kits used, together with the way in which the activities are expressed.

supplementary Table S11 Three-way factorial analysis of variance of the reactive oxygen species contents, defense enzyme activities and hormone contents, with cultivar, fungal strain and time as fixed factors, giving the degrees of freedom, F value and p value for every main effect and interaction.

supplementary Table S12 Degree, betweenness and closeness centrality of ERF1 and EIN3 in the b-locus-dependent transcription-factor networks of the two cultivars, with the rank and percentile of each value within its own network.

supplementary Table S13 TO-GCN level assignment and degree of every transcription factor in the two networks.

supplementary Table S14 FPKM expression matrix of the 2,933 transcription factors that make up the two networks, across all 60 RNA-seq libraries.

supplementary Data Sheet 1 Python scripts used for the breadth-first-search level assignment, the seed-node and correlation-cutoff sensitivity analyses, the network centrality calculations and the three-way factorial analysis of variance, with the input files and parameters documented in the header.

supplementary Figure S1 Expression profile of the seed gene (EREBP, SoffiXsponR570.02Dg300000) across all five time points (0, 1, 3, 5, and 7 dpi) under WT and mutant infection in both cultivars

supplementary Figure S2 Robustness of the TO-GCN level assignments. **(A, B)** Five alternative early-expressed transcription factors, taken from levels 2 and 3 of the original network, were used as seed nodes in **(A)** GT11-80 and **(B)** GT29. The complete network was recovered in every case, and the resulting level assignment was compared with the original one using Spearman's rank correlation coefficient. **(C, D)** Mean time of peak expression under wild-type infection for the transcription factors of each level in **(C)** GT11-80 and **(D)** GT29; marker area is proportional to the number of transcription factors in the level. The level of a transcription factor is correlated with the time at which its expression peaks, which is a check that does not depend on the seed node. The last levels contain very few transcription factors, so their mean values are imprecise.

supplementary Figure S3 Quality control of the 60 RNA-seq libraries. **(A)** Pearson correlation between all pairs of libraries, calculated on log2(FPKM + 1) values of 79,006 expressed genes. **(B)** Principal component analysis of the same data. **(C)** Distribution of correlation coefficients within groups of biological replicates and between different treatment groups.

supplementary Figure S4 Empirical distribution of the Pearson correlation coefficients of all transcription-factor pairs and derivation of the correlation cutoff. **(A, B)** Density of the coefficients under wild-type (W) and b-locus mutant (M) infection in **(A)** GT11-80 and **(B)** GT29. The shaded band marks the M0 window and the dotted line marks the W+ cutoff applied. **(C, D)** Proportion of transcription-factor pairs exceeding a given coefficient under wild-type infection in **(C)** GT11-80 and **(D)** GT29, with the 5% level indicated. The cutoffs were determined with the Cutoff module of the TO-GCN package and correspond to 4.07% and 4.30% of all pairs, respectively.

supplementary Figure S5 Sensitivity of the TO-GCN level assignments to the correlation cutoff. The network was rebuilt at progressively more stringent cutoffs in **(A)** GT11-80 and **(B)** GT29, and the resulting level assignment of each transcription factor was compared with the original assignment using Spearman's rank correlation coefficient (blue, left axis). The percentage of transcription factors still placed in the network is also shown (red, right axis). The dotted line marks the cutoff used in this study. Because a sparser network requires more breadth-first search steps, the number of levels increases with the cutoff and the absolute level numbers are rescaled; the relative temporal order, which is the feature interpreted in this study, is preserved. The test could only be carried out towards more stringent cutoffs, and the M0 window used to define the b-locus-dependent pairs was not varied.
