# Supplementary material for: Time-ordered co-expression network analysis reveals b-locus-mediated transcriptional cascades underlying sugarcane resistance to smut fungus (Sporisorium scitamineum)
Source: Front Plant Sci. 2026 Oct 16;17:1929311. doi: 10.3389/fpls.2026.1929311 (PMC13721741; doi:10.3389/fpls.2026.1929311)
Supplement: Supplementary file 1 [file DataSheet1.docx]

**Supplementary Material**

**Time-Ordered Co-expression Network Analysis Reveals b-Locus-Mediated Transcriptional Cascades Underlying Sugarcane Resistance to Smut Fungus (*Sporisorium scitamineum*)**

Xiu-Peng Song, Xiao-Qiu Zhang, Bing-Qiang Lu, Dong-Mei Huang, Yong-Jian Liang, Bao-Qing Zhang, Yu-Chi Deng, Dong-Mei Li, Yang-Rui Li, Qiang Liang*, Krishan K. Verma*, Mei-Xin Yan*

** Correspondence: liangqiangde@163.com; drvermakishan@gmail.com; yanmeixin@gxaas.net*

This file contains Supplementary Figures S1–S5 with their legends, followed by the legends of Supplementary Tables S1–S14 and Supplementary Data Sheet 1, which are supplied as separate data files.

**Supplementary Figures**

**Supplementary Figure S1.** Expression profile of the seed gene (EREBP, SoffiXsponR570.02Dg300000) across all five time points (0, 1, 3, 5, and 7 dpi) under WT and mutant infection in both cultivars.

**Supplementary Figure S2.** Robustness of the TO-GCN level assignments. (A, B) Five alternative early-expressed transcription factors, taken from levels 2 and 3 of the original network, were used as seed nodes in (A) GT11-80 and (B) GT29. The complete network was recovered in every case, and the resulting level assignment was compared with the original one using Spearman's rank correlation coefficient. (C, D) Mean time of peak expression under wild-type infection for the transcription factors of each level in (C) GT11-80 and (D) GT29; marker area is proportional to the number of transcription factors in the level. The level of a transcription factor is correlated with the time at which its expression peaks, which is a check that does not depend on the seed node. The last levels contain very few transcription factors, so their mean values are imprecise.

**Supplementary Figure S3.** Quality control of the 60 RNA-seq libraries. (A) Pearson correlation between all pairs of libraries, calculated on log2(FPKM + 1) values of 79,006 expressed genes. (B) Principal component analysis of the same data. (C) Distribution of correlation coefficients within groups of biological replicates and between different treatment groups.

**Supplementary Figure S4.** Empirical distribution of the Pearson correlation coefficients of all transcription-factor pairs and derivation of the correlation cutoff. (A, B) Density of the coefficients under wild-type (W) and *b*-locus mutant (M) infection in (A) GT11-80 and (B) GT29. The shaded band marks the M0 window and the dotted line marks the W+ cutoff applied. (C, D) Proportion of transcription-factor pairs exceeding a given coefficient under wild-type infection in (C) GT11-80 and (D) GT29, with the 5% level indicated. The cutoffs were determined with the Cutoff module of the TO-GCN package and correspond to 4.07% and 4.30% of all pairs, respectively.

**Supplementary Figure S5.** Sensitivity of the TO-GCN level assignments to the correlation cutoff. The network was rebuilt at progressively more stringent cutoffs in (A) GT11-80 and (B) GT29, and the resulting level assignment of each transcription factor was compared with the original assignment using Spearman's rank correlation coefficient (blue, left axis). The percentage of transcription factors still placed in the network is also shown (red, right axis). The dotted line marks the cutoff used in this study. Because a sparser network requires more breadth-first search steps, the number of levels increases with the cutoff and the absolute level numbers are rescaled; the relative temporal order, which is the feature interpreted in this study, is preserved. The test could only be carried out towards more stringent cutoffs, and the M0 window used to define the *b*-locus-dependent pairs was not varied.

**Supplementary Tables**

*The following supplementary tables and the Supplementary Data Sheet are provided as separate data files.*

**Supplementary Table S1.** Transcription factor co-expression pairs used to construct the susceptible GT11-80 TO-GCN.

**Supplementary Table S2.** KEGG enrichment results for *b*-locus-dependent genes across GT11-80 TO-GCN levels.

**Supplementary Table S3.** GO enrichment results for *b*-locus-dependent genes across GT11-80 TO-GCN levels.

**Supplementary Table S4.** Transcription factor co-expression pairs used to construct the resistant GT29 TO-GCN.

**Supplementary Table S5.** KEGG enrichment results for *b*-locus-dependent genes across GT29 TO-GCN levels.

**Supplementary Table S6.** GO enrichment results for *b*-locus-dependent genes across GT29 TO-GCN levels.

**Supplementary Table S7.** Primer sequences used for SYBR Green qPCR quantification of *S. scitamineum* *bE* gene.

**Supplementary Table S8.** Quality-control and mapping statistics of the 60 RNA-seq libraries, including raw and clean read numbers, Q20, Q30, GC content and mapping rates.

**Supplementary Table S9.** Validation parameters of the LC-MS/MS method used for phytohormone quantification, including MRM transitions, calibration ranges, limits of detection and quantification, recoveries, and the lowest measured group mean relative to the limit of quantification for each analyte.

**Supplementary Table S10.** Reaction principle, detection wavelength and definition of one unit of enzyme activity for each of the seven commercial assay kits used, together with the way in which the activities are expressed.

**Supplementary Table S11.** Three-way factorial analysis of variance of the reactive oxygen species contents, defense enzyme activities and hormone contents, with cultivar, fungal strain and time as fixed factors, giving the degrees of freedom, F value and p value for every main effect and interaction.

**Supplementary Table S12.** Degree, betweenness and closeness centrality of ERF1 and EIN3 in the *b*-locus-dependent transcription-factor networks of the two cultivars, with the rank and percentile of each value within its own network.

**Supplementary Table S13.** TO-GCN level assignment and degree of every transcription factor in the two networks.

**Supplementary Table S14.** FPKM expression matrix of the 2,933 transcription factors that make up the two networks, across all 60 RNA-seq libraries.

**Supplementary Data Sheet 1.** Python scripts used for the breadth-first-search level assignment, the seed-node and correlation-cutoff sensitivity analyses, the network centrality calculations and the three-way factorial analysis of variance, with the input files and parameters documented in the header.
