# Supplementary material for: Time-ordered co-expression network analysis reveals b-locus-mediated transcriptional cascades underlying sugarcane resistance to smut fungus (Sporisorium scitamineum)
Source: Front Plant Sci. 2026 Oct 16;17:1929311. doi: 10.3389/fpls.2026.1929311 (PMC13721741; doi:10.3389/fpls.2026.1929311)
Supplement: Supplementary file 2 [file SupplementaryFile1.docx]

**Supplementary Data Sheet 1**

Python scripts used for the breadth-first-search level assignment, the seed-node and correlation-cutoff sensitivity analyses, the network centrality calculations and the three-way factorial analysis of variance. The code below can be copied into a file named analysis_scripts.py and run with Python 3; input files and parameters are documented in the header.

```
#!/usr/bin/env python3
"""
Supplementary Data Sheet 1
Analysis scripts for the network and statistical analyses reported in this study.

Song X.-P. et al. Time-ordered gene co-expression network analysis of the
sugarcane-Sporisorium scitamineum interaction.

--------------------------------------------------------------------------
INPUT FILES (all provided as Supplementary Material with this article)
--------------------------------------------------------------------------
  Supplementary_table_S1_S_TFs_TOGCN.tsv    edge list, GT11-80 (71,964 pairs)
  Supplementary_table_S4_R_TFs_TOGCN.tsv    edge list, GT29    (31,977 pairs)
  Supplementary_table_S13_TF_level_assignment.xlsx   TO-GCN level of every TF
  Supplementary_table_S14_TF_expression_matrix.tsv   FPKM of the network TFs
  Supplementary_table_S11_three_way_ANOVA.xlsx       group means and SD used below

--------------------------------------------------------------------------
WHAT THIS SCRIPT REPRODUCES
--------------------------------------------------------------------------
  1. Breadth-first-search level assignment          -> Figures 5A and 6A
  2. Sensitivity to the choice of seed node          -> Supplementary Figure S2A, S2B
  3. Level versus measured time of peak expression   -> Supplementary Figure S2C, S2D
  4. Sensitivity to the correlation cutoff           -> Supplementary Figure S5
  5. Degree, betweenness and closeness centrality    -> Supplementary Table S12
  6. Three-way factorial analysis of variance        -> Supplementary Table S11

--------------------------------------------------------------------------
REQUIREMENTS
--------------------------------------------------------------------------
  Python >= 3.9, networkx >= 3.0, scipy >= 1.10, openpyxl >= 3.1
  (no other dependency; run time a few minutes on a standard laptop)

--------------------------------------------------------------------------
NOTES ON THE NETWORK
--------------------------------------------------------------------------
  The co-expression cutoffs were derived from the data with the Cutoff module
  of the TO-GCN package (Chang et al., 2019, PNAS 116:3091-3099), which selects
  the value beyond which the probability of exceeding it by chance falls below
  0.05.  The values applied were:

        positive co-expression under wild-type infection (W+)
              PCC > 0.93   in GT11-80
              PCC > 0.90   in GT29
        no correlation under mutant infection (M0)
              -0.5 <= PCC <= 0.5   in both cultivars

  The networks analysed in the paper are the sets of transcription factors that
  the breadth-first search reaches from the seed node: 2,539 TFs / 71,964 pairs
  in GT11-80 and 2,380 TFs / 31,977 pairs in GT29.

  Seed nodes:  GT11-80  SoffiXsponR570.02Dg300000  (EREBP)
               GT29     SoffiXsponR570.01Fg150100
"""

import csv, collections, itertools, math, statistics as st
import networkx as nx
from scipy.stats import spearmanr, studentized_range, f as f_dist

SEED = {"GT11-80": "02Dg300000", "GT29": "01Fg150100"}
EDGES = {"GT11-80": "Supplementary_table_S1_S_TFs_TOGCN.tsv",
         "GT29":    "Supplementary_table_S4_R_TFs_TOGCN.tsv"}


# ---------------------------------------------------------------- 1. network
def load_network(path):
    """Read an edge list and return an undirected graph with PCC as edge weight."""
    G = nx.Graph()
    with open(path, encoding="utf8", errors="replace") as fh:
        rd = csv.reader(fh, delimiter="\t")
        next(rd)                                    # header
        for row in rd:
            if len(row) < 4:
                continue
            try:
                pcc = float(row[3])                 # PCC under C1 (wild type)
            except ValueError:
                continue
            G.add_edge(row[0].strip(), row[2].strip(), pcc=pcc)
    return G


def bfs_levels(G, seed):
    """TO-GCN level assignment: level = shortest-path distance from the seed + 1."""
    level, current, k = {seed: 1}, {seed}, 1
    while current:
        k += 1
        nxt = set()
        for u in current:
            for v in G.neighbors(u):
                if v not in level:
                    level[v] = k
                    nxt.add(v)
        current = nxt
    return level


# --------------------------------------------------- 2. seed-node sensitivity
def seed_sensitivity(G, original_levels, n_alternatives=5):
    """Rebuild the ordering from alternative early-expressed seeds (levels 2 and 3)."""
    lv2 = sorted(g for g, l in original_levels.items() if l == 2)[:3]
    lv3 = sorted(g for g, l in original_levels.items() if l == 3)[:2]
    out = []
    for s in (lv2 + lv3)[:n_alternatives]:
        new = bfs_levels(G, s)
        shared = [g for g in new if g in original_levels]
        rho, _ = spearmanr([new[g] for g in shared],
                           [original_levels[g] for g in shared])
        out.append((s, original_levels[s], len(new), rho))
    return out


# ------------------------------------------- 3. level vs peak expression time
TIME_POINTS = [0, 1, 3, 5, 7]                      # days post inoculation


def peak_time_correlation(levels, expression_file, cultivar_tag):
    """Correlate the TO-GCN level with the time at which each TF peaks (wild type)."""
    with open(expression_file, encoding="utf8", errors="replace") as fh:
        rd = csv.reader(fh, delimiter="\t")
        header = next(rd)
        cols = {t: [] for t in TIME_POINTS}
        for j, name in enumerate(header):
            if not name.startswith("W"):            # W = wild type, T = mutant
                continue
            body = name[1:].split("_")[0]           # e.g. 01180 -> time 0, cultivar 1180
            for t in TIME_POINTS:
                if body.startswith(str(t)) and body[len(str(t)):] == cultivar_tag:
                    cols[t].append(j)
        lv, pk = [], []
        for row in rd:
            gene = row[0].replace("SoffiXsponR570.", "")
            if gene not in levels:
                continue
            profile = [st.mean(float(row[j]) for j in cols[t]) for t in TIME_POINTS]
            if max(profile) <= 1:                   # ignore genes that are not expressed
                continue
            lv.append(levels[gene])
            pk.append(TIME_POINTS[profile.index(max(profile))])
    rho, p = spearmanr(lv, pk)
    return rho, p, len(lv)


# ----------------------------------------------- 4. correlation-cutoff test
def cutoff_sensitivity(G, seed, original_levels, cutoffs):
    """Rebuild the network at more stringent cutoffs and compare the level order."""
    out = []
    for c in cutoffs:
        H = nx.Graph((u, v) for u, v, d in G.edges(data=True) if d["pcc"] >= c)
        if seed not in H:
            continue
        new = bfs_levels(H, seed)
        shared = [g for g in new if g in original_levels]
        rho, _ = spearmanr([new[g] for g in shared],
                           [original_levels[g] for g in shared])
        out.append((c, H.number_of_edges(), len(new), max(new.values()), rho))
    return out


# --------------------------------------------------------- 5. centrality
def centrality(G, seed, genes):
    """Degree, betweenness and closeness (exact), with the rank of each gene."""
    H = G.subgraph(nx.node_connected_component(G, seed)).copy()
    n = H.number_of_nodes()
    deg = dict(H.degree())
    btw = nx.betweenness_centrality(H, normalized=True)   # exact, deterministic
    clo = nx.closeness_centrality(H)

    def rank(d, g):
        return sorted(d.values(), reverse=True).index(d[g]) + 1

    res = {}
    for g in genes:
        if g in H:
            res[g] = dict(degree=deg[g], degree_rank=rank(deg, g),
                          betweenness=btw[g], betweenness_rank=rank(btw, g),
                          closeness=clo[g], closeness_rank=rank(clo, g), n=n)
    return res


# --------------------------------------------- 6. three-way factorial ANOVA
def three_way_anova(cell_mean, cell_sd, n=3):
    """
    Exact factorial ANOVA from the cell means and standard deviations of a
    balanced design.  cell_mean / cell_sd are dictionaries keyed by
    (cultivar, strain, time).  Returns F, df and p for every effect.
    """
    keys = list(cell_mean)
    levels = [sorted({k[i] for k in keys}) for i in range(3)]
    grand = sum(cell_mean.values()) / len(keys)
    sse = sum((n - 1) * cell_sd[k] ** 2 for k in keys)
    dfe = len(keys) * (n - 1)
    mse = sse / dfe
    N = len(keys) * n

    def marginal(dims, combo):
        sel = [k for k in keys if all(k[d] == c for d, c in zip(dims, combo))]
        return sum(cell_mean[k] for k in sel) / len(sel)

    effects = {"Cultivar": [0], "Strain": [1], "Time": [2],
               "Cultivar x Strain": [0, 1], "Cultivar x Time": [0, 2],
               "Strain x Time": [1, 2], "Cultivar x Strain x Time": [0, 1, 2]}
    out = {}
    for name, dims in effects.items():
        combos = list(itertools.product(*[levels[d] for d in dims]))
        ss = 0.0
        for combo in combos:
            eff = 0.0
            for r in range(len(dims) + 1):          # inclusion-exclusion
                for sub in itertools.combinations(range(len(dims)), r):
                    sd_ = [dims[i] for i in sub]
                    sc_ = [combo[i] for i in sub]
                    eff += (-1) ** (len(dims) - r) * (marginal(sd_, sc_) if sd_ else grand)
            ss += (N // len(combos)) * eff ** 2
        df = 1
        for d in dims:
            df *= len(levels[d]) - 1
        F = (ss / df) / mse
        out[name] = dict(df=df, error_df=dfe, F=F, p=float(1 - f_dist.cdf(F, df, dfe)))
    return out


def tukey_letters(means, mse, n=3, k=4, df=32, alpha=0.05):
    """Compact letter display from Tukey's HSD, used for the letters in Figures 2-4."""
    hsd = studentized_range.ppf(1 - alpha, k, df) * (mse / n) ** 0.5
    N = len(means)
    ns = [[abs(means[i] - means[j]) < hsd for j in range(N)] for i in range(N)]
    order = sorted(range(N), key=lambda i: -means[i])
    groups = []
    for i in order:
        if not any(all(ns[i][j] for j in g) for g in groups):
            groups.append([j for j in order if ns[j][i]])
    groups = [set(g) for g in groups]
    groups = [g for g in groups if not any(g < h for h in groups)]
    uniq = []
    for g in groups:
        if g not in uniq:
            uniq.append(g)
    uniq.sort(key=lambda g: -max(means[i] for i in g))
    labels = [""] * N
    for gi, g in enumerate(uniq):
        for i in g:
            labels[i] += chr(97 + gi)
    return ["".join(sorted(s)) for s in labels], hsd


# --------------------------------------------------------------- driver
if __name__ == "__main__":
    ERF1, EIN3 = "01Cg002500", "01Bg347100"
    for cultivar, tag in (("GT11-80", "1180"), ("GT29", "29")):
        G = load_network(EDGES[cultivar])
        levels = bfs_levels(G, SEED[cultivar])
        print(f"\n=== {cultivar} ===")
        print(f"  network: {len(levels)} TFs, "
              f"{G.subgraph(levels).number_of_edges()} pairs, "
              f"{max(levels.values())} levels")

        print("  seed-node sensitivity (Supplementary Figure S2A, S2B)")
        for s, l, recovered, rho in seed_sensitivity(G, levels):
            print(f"    seed {s} (level {l}): {recovered} TFs recovered, rho = {rho:.3f}")

        rho, p, m = peak_time_correlation(
            levels, "Supplementary_table_S14_TF_expression_matrix.tsv", tag)
        print(f"  level vs peak expression time (Supplementary Figure S2C, S2D): "
              f"rho = {rho:.3f}, p = {p:.2e}, n = {m}")

        base = 0.93 if cultivar == "GT11-80" else 0.90
        grid = [round(base + 0.01 * i, 2) for i in range(6)]
        print("  cutoff sensitivity (Supplementary Figure S5)")
        for c, e, kept, lv, rho in cutoff_sensitivity(G, SEED[cultivar], levels, grid):
            print(f"    cutoff {c:.2f}: {e:6d} pairs, {kept:5d} TFs, "
                  f"{lv:2d} levels, rho = {rho:.3f}")

        print("  centrality (Supplementary Table S12)")
        for g, v in centrality(G, SEED[cultivar], [ERF1, EIN3]).items():
            name = "ERF1" if g == ERF1 else "EIN3"
            print(f"    {name}: degree {v['degree']} (rank {v['degree_rank']}/{v['n']}), "
                  f"betweenness rank {v['betweenness_rank']}, "
                  f"closeness rank {v['closeness_rank']}")
```
